# Deletion of exchange proteins directly activated by cAMP (Epac) causes defects in hippocampal signaling in female mice

**DOI:** 10.1371/journal.pone.0200935

**Published:** 2018-07-26

**Authors:** Reidun Aesoy, Haruna Muwonge, Kathrine S. Asrud, Misbah Sabir, Solveig L. Witsoe, Ronja Bjornstad, Reidun K. Kopperud, Erling A. Hoivik, Stein Ove Doskeland, Marit Bakke

**Affiliations:** 1 Department of Clinical Science, University of Bergen, Bergen, Norway; 2 Department of Biomedicine, University of Bergen, Bergen, Norway; 3 Department of Clinical Medicine, University of Bergen, Bergen, Norway; University of Modena and Reggio Emilia, ITALY

## Abstract

Previous studies demonstrate essential roles for the exchange proteins directly activated by cAMP 1 and 2 (Epac1 and Epac2; here collectively referred to as Epac) in the brain. In the hippocampus, Epac contributes to the control of neuronal growth and differentiation and has been implicated in memory and learning as well as in anxiety and depression. In the present study we address the hypothesis that Epac affects hippocampal cellular responses to acute restraint stress. Stress causes activation of the hypothalamus-pituitary-adrenal (HPA)-axis, and glucocorticoid receptor (GR) signaling is essential for proper feedback regulation of the stress response, both in the brain and along the HPA axis. In the hippocampus, GR expression is regulated by cAMP and the brain enriched micro RNA miR-124. Epac has been associated with miR-124 expression in hippocampal neurons, but not in regulation of GR. We report that hippocampal expression of Epac1 and Epac2 increased in response to acute stress in female wild type mice. In female mice genetically deleted for Epac, nuclear translocation of GR in response to restraint stress was significantly delayed, and moreover, miR-124 expression was decreased in these mice. Male mice lacking Epac also showed abnormalities in miR-124 expression, but the phenotype was less profound than in females. Serum corticosterone levels were slightly altered immediately after stress in both male and female mice deleted for Epac. The presented data indicate that Epac1 and Epac2 are involved in controlling cellular responses to acute stress in the mouse hippocampus and provide novel insights into the underlying transcriptional and signaling networks. Interestingly, we observe sex specific differences when Epac is deleted. As the incidence and prevalence of stress-related diseases are higher in women than in men, the Epac knockout models might serve as genetic tools to further elucidate the cellular mechanisms underlying differences between male and female with regard to regulation of stress.

## Introduction

The hypothalamus-pituitary-adrenal (HPA) axis is activated in response to stress, resulting in the release of glucocorticoids (GCs) from the adrenal cortex. Excess of GCs has adverse effects on homeostasis, and an intricate negative feedback system has evolved to control the levels of circulating GCs. The major GC effector in stressful situations is the glucocorticoid receptor (GR; systematic name Nr3c1), which regulates target genes along the HPA axis as well as in the hippocampus in response to increased GC [1]. The hippocampus is an essential regulator of HPA axis activity [2] and disruption of adult hippocampal neurogenesis causes a stress related phenotype [3]. Moreover, normal hippocampal expression of GR is a prerequisite for intact HPA axis activity, and forebrain specific deletion of GR leads to a hyperactive HPA axis and depression-like behavior in mice [4].

The signaling molecule cAMP is fundamental for proper control of the HPA axis [5], and also regulates a number of hippocampal functions, including responses to stress [6, 7]. The mammalian cAMP binding proteins include the well-studied cAMP dependent protein kinase (PKA) and the less studied Exchange Proteins directly Activated by cAMP (Epac1 and Epac2; also known as Rap Guanine Nucleotide Exchange Factors 1 and 2; here collectively referred to as Epac) discovered in the late 1990’s [8–10]. A number of cAMP-mediated physiological processes first attributed solely to PKA have later been demonstrated to also involve Epac [11]. Epac1 and Epac2 are encoded by *Rapgef3* and *Rapgef4*, respectively, and whereas *Rapgef3* is transcribed in most tissues leading to near ubiquitous expression of Epac1 [12], three transcripts produced from *Rapgef4* give rise to the three proteins Epac2A, Epac2B and Epac2C that are expressed in a strict tissue-specific pattern. Epac2A is expressed predominantly in brain, pituitary and endocrine pancreas, Epac2B in steroidogenic cells of the adrenal gland and testis, and in the endocrine pancreas, while Epac2C has so far only been found in liver [13–16]. The Epac2 isoforms are gradually shortened from the N-terminus and arise from epigenetically controlled alternative promoters [14–16].

Both Epac1 and Epac2 are expressed in the adult mouse brain, but Epac2 at a higher level than Epac1 with abundant expression in cortex, hippocampus and thalamus [10, 17]. Gene targeting in mice has demonstrated multiple roles for Epac in the brain [18], and whereas certain phenotypes are manifested by single gene knockout strategies [19–22], some phenotypes are manifested only after deletion of both Epac1 and Epac2 [23]. Whether this latter observation is a result of redundant functions of the two proteins or because Epac1 and Epac2 have different functions in a given physiological process is still unknown. Interestingly, detailed analyses of several Epac knockout models reveal that deletion of either Epac1 or Epac2, or both, does not produce gross anatomical or physiological abnormalities, but that various phenotypes are provoked upon exposure of the mice to challenging situations [20, 21, 23–27]. In the hippocampus, neuroanatomy and synaptic structures appear normal in mice deleted for Epac2 (Epac2^-/-^) or both Epac1 and Epac2 (Epac1/2^-/-^), but various molecular processes are affected leading to defect neurogenesis, deficits in long-term plasticity, spatial learning and social interactions, and increased anxiety and depression [19–21, 23].

GR is a nuclear receptor type of transcription factor that upon ligand binding translocates from the cytoplasm to the nucleus where it interacts with promoters of target genes to regulate gene expression. In addition to ligand binding, GR activity and cellular sub-localization is also determined by posttranslational modifications [28]. In the mouse and rat hippocampus, such intracellular redistribution typically occurs in response to stress with increased GR protein levels in the nuclear fraction [29–32]. The cAMP and GR signaling pathways integrate to modify gene expression [33–35]. For instance, in the hippocampus, the transcription factor Ngfi-A (Nerve growth factor induced clone-A; also called Early Growth Response 1; Egr1), which is an essential regulator of the gene encoding GR (*Nr3c1*), is induced by cAMP [36]. Clearly suggesting a pathophysiological relevance for the GR-cAMP interaction is the finding that neonatal maternal neglect causes abnormal serotonin and cAMP signaling, with subsequent increased DNA methylation at the Ngfi-A binding site in the *Nr3c1* promoter, resulting in decreased GR expression and a depressed like phenotype in rats [37]. GR expression is under complex regulatory control, and the GR mRNA contains binding sites for multiple microRNAs, including the brain enriched microRNA miR-124 [38]. MiR-124 is abundantly expressed in the mouse brain [39] and associated with multiple brain functions, including stress [40, 41]. *In vitro* based experimental data indicate that miR-124 interacts with the 3’untranslated region of the mRNA encoding GR, leading to decreased GR expression [38, 42]. It has also been suggested that miR-124 influences GR activity indirectly via effects on phosphodiesterase 4B again linking cAMP signaling and GR function [42].

The hippocampal phenotypes (i.e. deficits in long-term plasticity, spatial learning and social interactions) observed in Epac1/2^-/-^ mice have been proposed to arise partly from a disturbed balance between miR-124 and Ngfi-A [23]. Based on the roles of Ngfi-A as an important regulator of GR expression, we hypothesized that mice deleted for Epac1, Epac2 or both factors would present with irregular stress and GR responses. We report that, immediately after restraint stress, both male and female mice presented with slightly altered corticosterone levels in the absence of Epac. However, only female knockout mice exhibited delayed GR nuclear translocation after stress, and also, only in female wt mice did the hippocampal mRNA levels of Epac1 and Epc2 increase after stress. Finally, we observed a consistent reduction in miR-124 expression in female mice lacking Epac. The presented results therefore indicate that Epac signaling in the hippocampus differ in male and female mice in response to acute stress.

## Materials and methods

### Animals

Mice used in this study were on a C57Bl/6 genetic background. The origin of the C57BL/*6*JBomTac mice is described at http://www.taconic.com/mouse-model/b6jbom. The knockout models for Epac1 (Epac1^-/-^) and Epac2 (Epac2^-/-^) have been described elsewhere [43, 44]. Of note, the Epac2^-/-^ model used in this study is deleted for all Epac2 isoforms (*i*.*e*. Epac2A, Epac2B and Epac2C). Mice deleted for both Epac1 and Epac2 (Epac1/2^-/-^) were generated by crossing of Epac1^-/-^ and Epac2^-/-^ mice. The knockout models presented no gross phenotypes under standard animal housing. The mice were housed 2–5 animals/cage and bred at the animal facility at Haukeland University Hospital and kept on a 12:12-h light-dark cycle (lighting of 150 lux and lights on at 06:00 hr), RT 22 ± 1°C, and humidity of 55% ± 5%. All mice used in this study were littermates, or from different litters bred in the same room. The Animal Care and Use Programs at University of Bergen are accredited by AAALAC international and by the Norwegian Food Authority, and all animal handling and experiments involving animals at the facility are in accordance with the legislation and regulations of the Norwegian Animal Research Authority (under the Norwegian Food Authority). The experiments involving mice or biological samples from mice in the current study were specifically evaluated and approved by the Norwegian Animal Research Authority (FOTS project numbers 20135060 and 20158111).

### Restraint stress experiments

Wild type (wt), Epac1^-/-^, Epac2^-/-^ and Epac1/2^-/-^ mice (8–12 weeks of age) were either subjected to restraint stress for 30min or left undisturbed in their home cages. Restraint stress was conducted by placing the mouse in a ventilated tube (CODA^TM^ holder, Kent Scientific Co) for a time-period of 30min. Upon completion of the restraint, the mice were either euthanized immediately (0h recovery) or given a recovery time of 30min or 2h in their home cages, and subsequently euthanized. The mice were randomly assigned to the different groups and the restraint experiments were conducted between 12:00 pm and 2:00 pm in isolation from other mice. No adverse events were reported.

### Histological analyses

To examine the morphology of the hippocampus, hippocampal tissue sections were stained with Hematoxylin and Eosin (H&E), and visualized with a Leica DMLB light microscope under a 10X objective. The images shown were acquired with a Leica DC 300 camera (Leica Microsystems AG).

### Corticosterone assay

Mice were exposed to stress as described and euthanized by CO_2_ between 2:00 and 3:00 pm. The mice were euthanized in a CO_2_ chamber and only exposed to the gas until they stopped breathing (about 2 min) in order to minimize any artificial elevation of corticosterone by the euthanization procedure [45]. In rats, this procedure exerts minimal distress to the animals [45] and does not significantly increase corticosterone levels in trunk blood compared to termination by decapitation [46]. Similar studies comparing termination by CO_2_ and decapitation in mice have not been published, but the corticosterone levels detected in unstressed male and female mice after termination with CO_2_ in the current study are similar to those observed when decapitation is carried out on mice [47, 48]. Trunk blood was collected into BD Microtainer SST tubes (#365968, BD), centrifuged at 2000xg at RT for 15 min. Corticosterone levels were determined by a corticosterone Elisa kit (cat. # EIA-5186, DRG Instruments GmbH, Germany). Samples were randomized and run in duplicates. All procedures were performed according to the manufacturer’s instructions.

### Immunohistochemistry (IHC)

Intact brain tissue to be used for IHC was immediately post-fixed in 4% paraformaldehyde (4% PFA, 3X PBS, pH 7.2) for 24 h. After subsequent overnight dehydration in 70% EtOH the tissue was embedded in paraffin and later sectioned in 15 μM coronal sections and stored at 4°C until further analysis. The paraffin embedded sections were deparaffinized in xylene (2 x 3 min) and rehydrated in a graded series of ethanol (100% ethanol for 2 x 3 min, 95% ethanol for 3 min, 70% ethanol for 3 min and Milli-Q water for 2 x 2 min). Antigen retrieval using citrate buffer (10 mM sodium citrate, pH 6.0) was done at 98°C for 20 min. The slides were cooled in cold running tap water for 10 min, and incubated in blocking buffer (1X PBS (pH 7.4), 10% normal goat serum, 0.3% Triton™ X-100) for 2h at RT in a moist chamber with gentle shaking, followed by o/n incubation with a polyclonal antibody against GR (Cat. # sc-8992 (H300), Santa Cruz Biotechnology, 1:50 dilution) in a moist chamber. Slides were then rinsed in 1X PBS (3 x 5 min) on a shaking platform, before incubation with a secondary antibody solution (Alexa Fluor^®^ 594 goat anti-rabbit IgG (H+L), Cat #1205993, Life Technologies; 1:200 dilution in 1X PBS) for 2h in the dark at RT and gentle shaking. Slides prepared for image acquisition at 60X magnification were co-stained with DAPI reagent (Prolong^®^ Diamond Antifade Reagent with DAPI, Cat. # P36962, Life Technologies) to visualize the DNA in the nucleus. At least 12h after application of the mounting medium, the slides were analyzed by fluorescence microscopy.

### Image processing and data analysis

Tissue sections to be analyzed for intensity of GR staining were examined using an Axioplan 2 Imaging-e microscope (Carl Zeiss, Germany) with a 10X objective, and a Zeiss Axiocam HR digital camera (Carl Ziess, Germany) to capture the images. A series of coronal brain sections through the septotemporal axis of the hippocampus (Bregma -1.28mm to -2.12mm) was obtained per animal. Immunofluorescence intensity of the sections was analyzed using ImageJ 1.48v (Research Service Branch, National Institutes of Health, Bethesda, MD; available at: https://imagej.nih.gov/ij/) by generating a region of interest (ROI) of GR stained nuclei in the Cy3 channel at 10X magnification and measuring the difference between the mean grey value in the ROI from that of the background on each slide. Positive GR immunofluorescence was observed across all the layers (strata) of the hippocampus. However, quantification for GR immunofluorescence intensity was only done for regions containing pyramidal ((stratum pyramidale for the cornu ammonis (CA) 1 and CA3) and granular (stratum granulosum for the dentate gyrus (DG)) neuronal cell bodies. Only nuclei that were double-labeled for both DAPI and GR were quantified. At least 3 animals per group (12 mice per genotype) were analyzed for each experimental condition. Measurements were derived from at least 3 sections per animal. For each section, immunofluorescence intensity for DG, CA1, and the CA3 regions was determined. Image cropping and resizing was performed using Adobe Photoshop CS5 (San Jose, CA). 60X images were examined with a Nikon Te 2000-e microscope, and captured with a Nikon Digital Sight DS-U1 camera.

### Dexamethasone suppression test

Mice (8–12 weeks of age) received an intraperitoneal injection of the synthetic GR agonist dexamethasone (0.1mg/kg) (D4902, Sigma Aldrich) in 2% EtOH by volume of PBS) or buffered saline (controls; 2% EtOH by volume of PBS), and culled after 6h. All injections were administered between the hours of 08:00 and 09:00 am. Trunk blood was collected into BD Microtainer SST tubes (BD, Cat. #365968), centrifuged at 2000xg at RT for 15 min, and the samples kept at -80°C. Corticosterone levels were determined by a corticosterone Elisa kit (Cat. #EIA-5186, DRG Instruments GmbH, Germany). Animals were randomly assigned to the treatment groups, and no adverse events were reported during the experimental procedures.

### RNA isolation and qPCR

Hippocampus were excised from the intact mouse brain and immediately snap-frozen in liquid nitrogen. RNA was isolated from the hippocampus using the GenElute Mammalian Total RNA Miniprep Kit (Sigma-Aldrich, Cat. #RTN70-1KT). For qPCR of mRNA, 500 ng total RNA was reverse transcribed using the iScript cDNA synthesis kit from BioRad (California, USA, Cat. #170–8891). The qPCR reactions had a total volume of 10 μl containing 5 μl SYBR Green Supermix (BioRad Ca. USA, Cat. #170–8882), cDNA template (10 ng) with a final concentration of 250 nM of forward and reverse primer. qPCR was performed on a Roche Light Cycler 480 with the following cycling conditions: 1 cycle for 5min at 95 °C, and 45 cycles for 10s at 95 °C, 10s at 60 °C and 10s at 72 °C. Relative mRNA expression was determined using the ΔΔC_T_ method with Sdha (Succinate dehydrogenase complex, subunit A,) and Ppib (Peptidylprolyl Isomerase) as reference genes [49–51]. For qPCR of miR-124, the qScript miRNA cDNA synthesis kit from Quanta Biosciences (Gaithersburg, US, Cat. #95107–100) was used. Micro RNA was converted into cDNA starting from 500 ng total RNA that first was polyadenylated following the manufactures instructions. MiR-124 qPCR were carried out in a final volume of 10 μL; 2X PerfeCTa^®^ SYBR^®^ Green Supermix (5 μL), cDNA template (5 ng), PerfeCTa^®^ microRNA Assay Primers (200nM) and PerfeCTa^®^ Universal PCR Primer (200nM) (Quanta BioSciences, Inc.) qPCR was run on a Roche Light Cycler 480 at the following cycling conditions: 1 cycle for 2min at 95°C, 40 cycles for 5s at 95°C, 15s at 60°C and 15s at 70°C. Relative gene expression of miR-124 was determined as described [52] with Snord47, Snord66 and Rnu6 used as reference small nuclear RNAs. All qPCR reactions were run on cDNA synthesized from mRNA pooled from hippocampus from 4–7 mice. qPCR reactions were run in triplicates in three separate experiments. The size of the amplicons (PCR-products) was confirmed by running 1μl of the reaction-solution gained from the qPCR on a 2% agarose gel (data not shown). See S1 Table for primer sequences. The amount of RNA extracted from individual hippocampus was at a low boundary of detection and hippocampus from 4–7 mice were therefore pooled before further analyses. It can be argued that pooling of RNA samples from different individuals reduces the sensitivity and that information about the variability between members of the same group is lost. However, reducing sample variation by pooling will limit biological noise and better reflect potential differences between groups of the different genotypes.

### Statistics

Data sets were analyzed with Prism 6 (GraphPad) software (SanDiego, CA, USA). One-way analysis of variance (ANOVA) with Tukey’s adjustment for multiple comparisons, Two-way ANOVA with Tukey’s or Dunnett`s adjustment for multiple comparisons or a Two-tailed Unpaired t test with Welch`s correction was used for multiple comparisons of data presented as indicated in the figure legends. The degrees of freedom, F- and p-values for the different pair-wise comparisons are also provided in the figure legends. p≤0.05 was considered statistically significant.

## Results

### Circulating corticosterone levels are slightly altered in the absence of Epac

**Fig 1:**
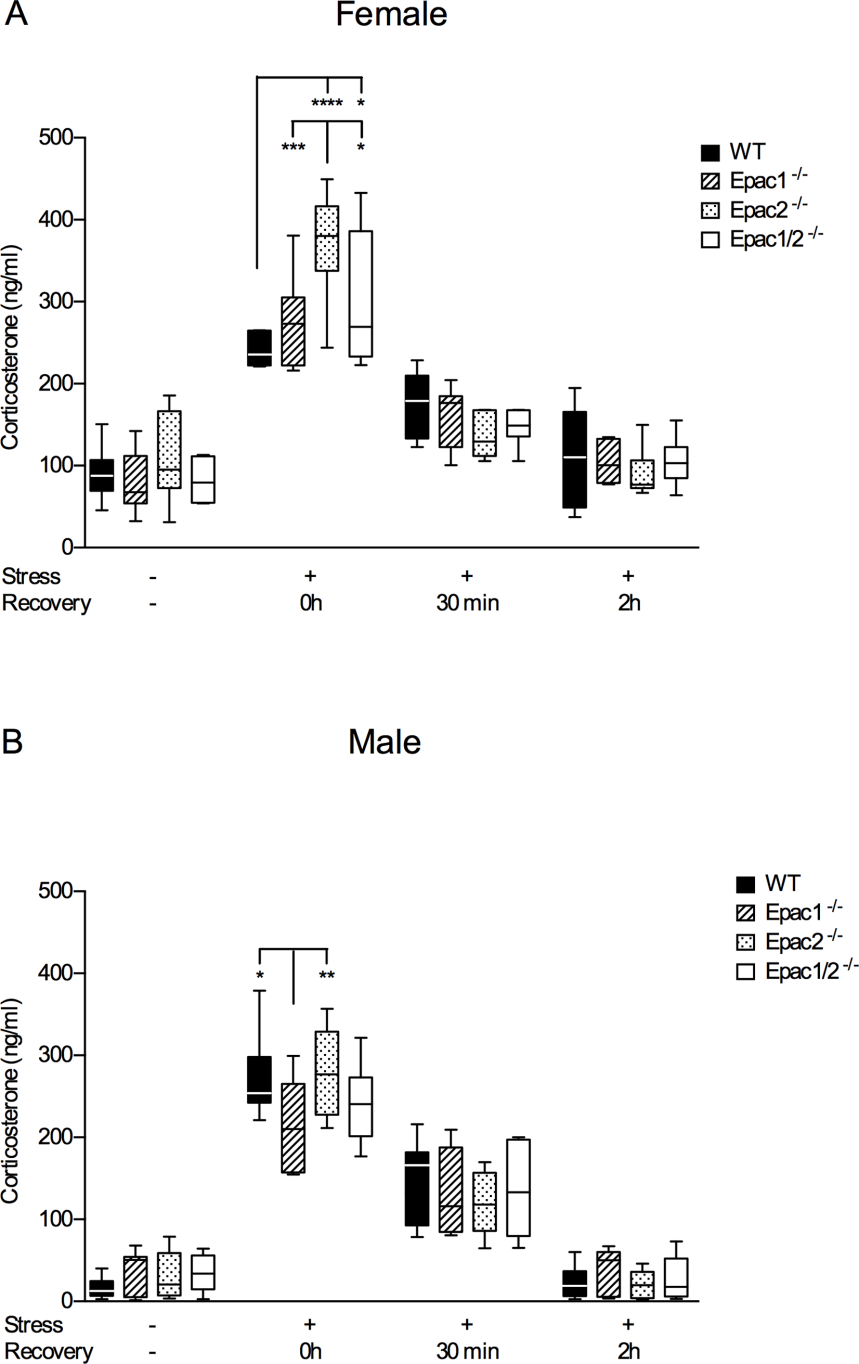
Serum corticosterone levels are disturbed immediately after restraint stress in the absence of Epac. Female **(A)** and male **(B)** wt, Epac1^-/-^, Epac2^-/-^ and Epac1/2^-/-^ mice were kept at standard housing conditions (-) or exposed to 30min restraint stress (+). Mice exposed to stress were culled either immediately after the stressor (0h), or after recovery from the stress for 30min or 2h, and trunk blood collected. Serum corticosterone levels were determined by ELISA. The corticosterone levels are shown as average ±SD. Two-way ANOVA with Tukey’s adjustment for multiple comparisons was used for statistical analysis. *p≤0.05,**p≤0.01, ***p<0.001 and ****p<0.0001. n = 7–9 mice per group. F-statistics (F(DFn, DFd)) for the female group: Interaction F(9,102) = 3.713, p = 0.0005 and male group: Interaction F(9,108) = 1.759, p = 0.0846.

To begin to determine the possible roles for hippocampal Epac in stress responses, male and female wt and Epac1^-/-^, Epac2^-/-^ and Epac1/2^-/-^ mice were exposed to acute stress by 30min restraint in ventilated plastic tubes. This psychological stressor produces fear without causing direct threat to wellbeing, and is relayed through limbic forebrain circuits, including the hippocampus [53]. Following restraint, the mice were either culled immediately or returned to their home cages for 30min or 2h recovery before culling. In agreement with established knowledge [1], a robust and transient increase in circulating corticosterone levels was observed in both sexes and all genotypes after exposure to restraint stress, and upon recovery for 30min or 2h, the corticosterone levels declined similarly in all groups (Fig 1 and S2 Table). Male mice exhibited lower levels of corticosterone than wt females in the absence of stress (15.3 ± 12.6 male wt mice vs. 90.6 ± 31.2 female wt mice, p = 0.0001, Two-tailed Unpaired t-test with Welch`s correction, F-statistics (F(DFn, DFd)) F(7,7) = 6.154, p = 0.0286). This sex difference has previously been observed [47, 48], but is not consistently reported in the literature [54]. We next determined whether deletion of Epac1, Epac2 or both factors affected the levels of circulating corticosterone in each treatment group. Female and male mice were analyzed separately (Fig 1). In both females and males, genotypic effects were found in the groups exposed to 30min stress and no recovery, whereas no effects of Epac deletions were evident in the other groups (Fig 1). In females, corticosterone levels were significantly higher in Epac2^-/-^ and Epac1/2^-/-^ mice than in wt mice, and the corticosterone levels of female Epac2^-/-^ mice were also elevated compared to Epac1^-/-^ and Epac1/2^-/-^ mice immediately after 30min of restraint stress (Fig 1A). In contrast, in males, the corticosterone levels of Epac1^-/-^ mice were slightly reduced compared to wt and to Epac2^-/-^ male mice (Fig 1B). However, male Epac2^-/-^ and Epac1/2^-/-^ mice did not differ from male wt mice (Fig 1B). The dexamethasone suppression test indicated normal GC feedback control at the pituitary level in the absence of Epac (S1 Fig).

### Epac1 and Epac2 mRNA levels are increased in the hippocampus of stressed female mice

Histological inspection revealed no apparent abnormalities in hippocampal anatomical structures in female or male Epac1^-/-^, Epac2^-/-^ and Epac1/2^-/-^ mice (S2 Fig). These findings are in accordance with previous studies reporting normal hippocampal morphology and synaptic structures in male Epac1/2^-/-^ mice [23] and male Epac2^-/-^ mice [19, 20]. Analyses of Epac1 and Epac2 mRNA levels demonstrated that Epac1 and Epac2 levels are not increased in a compensatory manner in the hippocampus of the opposite knockout model (S3 Fig). This is agreement with existing literature on hippocampus [21] and heart [55].

**Fig 2:**
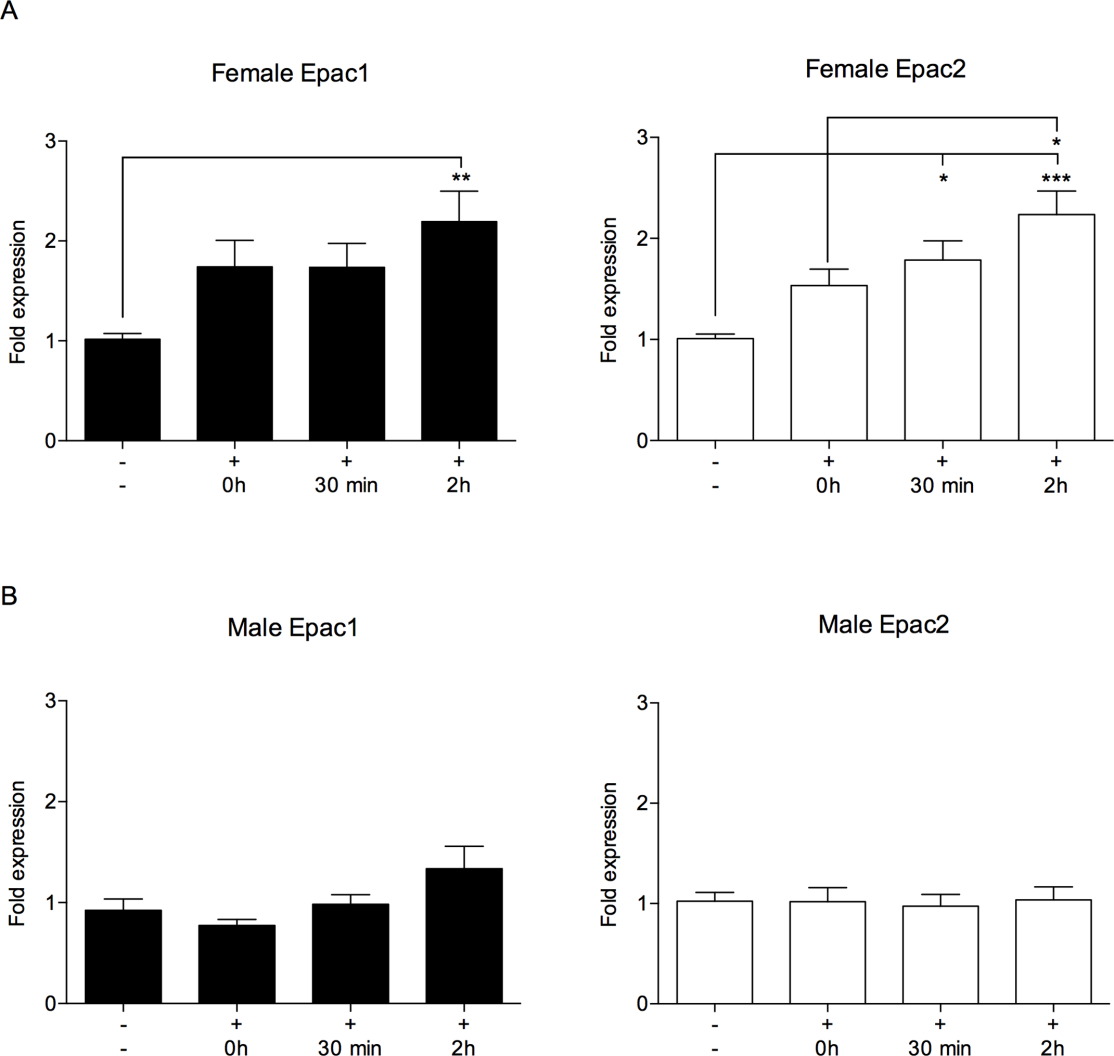
Epac1 and Epac2 mRNA levels are increased in wild type female mice following restraint stress. Female (**A**) and male (**B**) wt mice were kept at standard housing conditions (-) or exposed to 30min of restraint stress (+). The hippocampus was dissected out after the mice had been culled immediately after the stressor (0h), or after recovery from the stress for 30min or 2h. qPCR analyses were performed to determine the mRNA levels of Epac1 (black bars) and Epac2 (white bars). The qPCR values were normalized to the expression of the housekeeping genes Sdha and Ppib, and are shown as average of relative fold change ±SEM of three independent experiments performed in triplicates. Epac1 and Epac2 expression, and males and females, were analyzed separately. One-way ANOVA with Tukey’s adjustment for multiple comparisons was used for statistical analysis. *p≤0.05, **p≤0.01, ***p≤0.001. n = 7–11 mice per group. F-statistics (F(DFn, DFd)) Female Epac1: F(3, 40) = 4.217, p = 0.0111, Female Epac2: F(3, 34) = 7.649, p = 0.0005, Male Epac1: F(3, 29) = 3.286, p = 0.0347 and Male Epac2: F(3, 30) = 0.05371, p = 0.9833.

Male Epac1/2^-/-^ mice have reduced ability to cope with social stress in mice [23]. To determine whether hippocampal Epac expression is changed as a consequence of acute stress exposure, we analyzed Epac1 and Epac2 mRNA levels in female (Fig 2A) and male (Fig 2B) wt mice by qPCR. Epac1 and Epac2 mRNA levels increased in female mice after acute stress combined with recovery (at 2h of recovery for Epac1, and at 30min and 2h of recovery for Epac2). No equivalent increase in Epac expression was observed in male wt mice (Fig 2B). These results further supported roles for Epac in the acute stress response, and also indicate that these factors might have different roles in female and male hippocampus during stress.

### Female Epac1/2^-/-^ mice exhibit a delayed GR response after restraint stress

**Fig 3:**
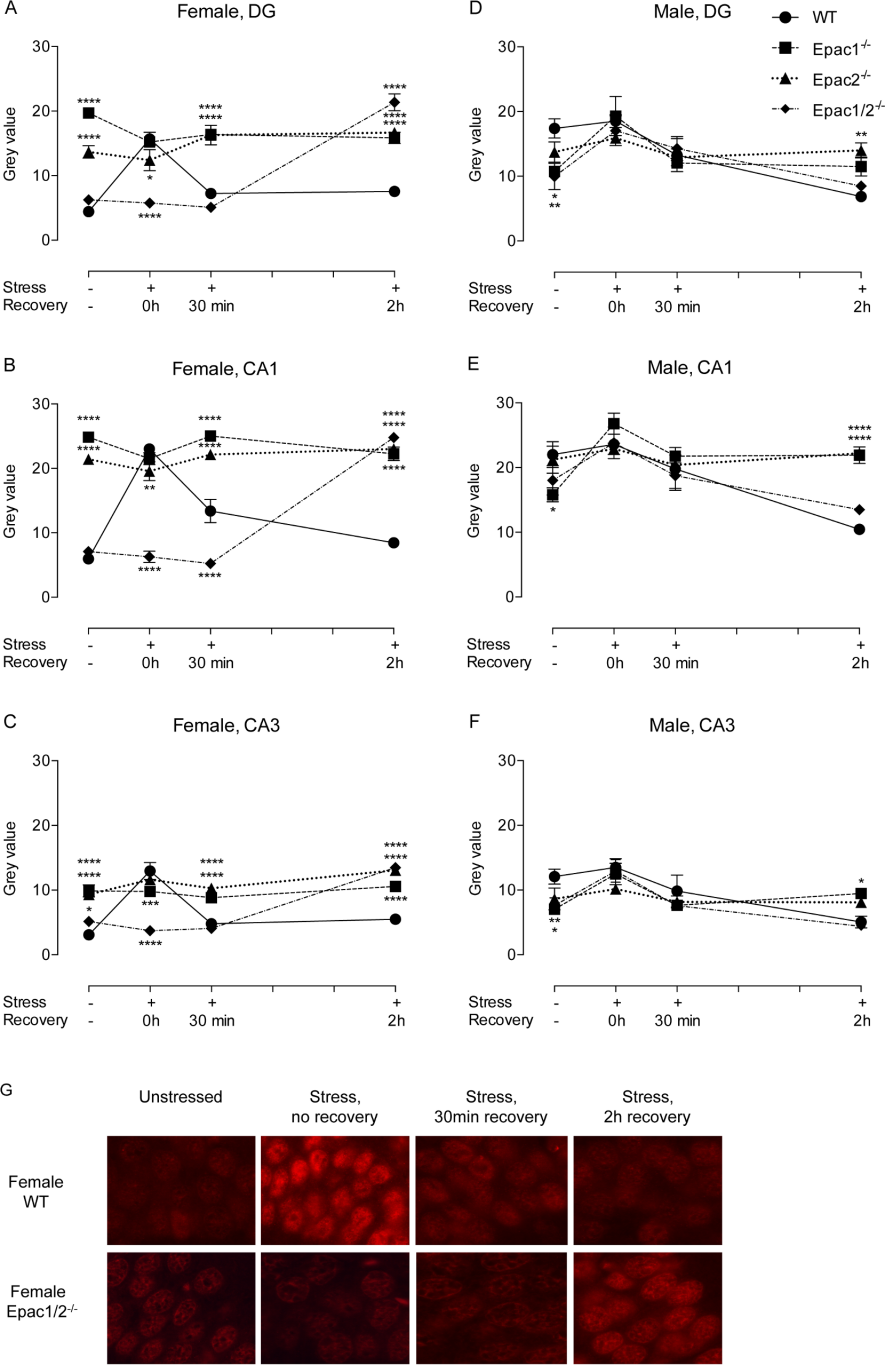
GR translocation is delayed in female Epac1/2^-/-^ mice. Paraffin-embedded brain sections were prepared from female (A-C, G) and male (D-F) wt, Epac1^-/-^, Epac2^-/-^ and Epac1/2^-/-^ mice kept at standard housing conditions (-) or exposed to 30min restraint stress (+). The hippocampus was dissected out after the mice had been culled immediately after the stressor (0h), or after recovery from the stress for 30min or 2h. Paraffin sections were subjected to IHC using a GR-specific antibody, and GR immunofluorescence was quantified using Image J software. Data are presented as average ±SEM gray values recorded from the DG, CA1, and CA3 regions as indicated. Two-way ANOVA with Dunnett’s adjustment for multiple comparisons was used to determine differences between genotypes in each treatment group in each region. *p≤0.05, **p≤0.01, ***p≤0.001 and ****p≤0.0001 wt mice compared to Epac1^-/-^, Epac2^-/-^ and Epac1/2^-/-^ mice (same timepoint). n = 3 mice per group, and for each mouse, 3 sections of the hippocampus where quantified for GR staining. F-statistics (F(DFn, DFd)) Female DG; Interaction: F(9, 128) = 35.68, p<0.0001, Female CA1; Interaction: F(9, 128) = 68.28, p<0.0001, Female CA3; Interaction: F(9, 128) = 26.84, p<0.0001, Male DG; Interaction: F(9, 131) = 2.861, p = 0.0041, Male CA1; Interaction: F(9, 131) = 4.433, p<0.0001, Male CA3; Interaction: F(9, 131) = 2.754, p = 0.0056. G) Representative GR IHC staining of the CA1 region of wt and Epac1/2^-/-^ female mice. Paraffin-embedded coronal brain sections (15μm) were stained with a GR-specific antibody and visualized under a 60X objective of the Nikon Te 2000-e microscope with a TRITC fluorescent light filter.

To determine whether deletion of Epac changed nuclear localization of GR in hippocampal sub-regions in response to stress, we performed IHC followed by quantification of GR staining in neuronal cell soma across the pyramidal layers of CA1 and CA3, and the granular layer of DG, regions in which stress induces dynamic changes in GR localization [56]. GR staining intensities recorded within each treatment group in the different hippocampal regions of wt mice were compared against staining intensity in the respective hippocampal regions and treatment group of Epac1^-/-^, Epac2^-/-^ and Epac1/2^-/-^ mice (Fig 3). In Table 1, the same recordings were statistically analyzed with regard to changes in staining intensities in response to different treatments in each brain region and genotype. Female and male mice were analyzed separately. Representative images of the GR IHC analyses are shown in S4 Fig (females) and S5 Fig (males). Co-staining with DAPI confirmed nuclear staining of GR after stress (S6 Fig).

**Table 1:** GR protein immunofluorescence is shown as mean Gray values ±SEM for female and male mice in the different genotype and treatment groups. Gray values monitored in each brain region (DG, CA1 and CA3) from unstressed (No) and stressed (0h, 30min and 2h recovery) mice were compared and significance determined by One-way ANOVA with Tukey’s adjustment for multiple comparisons. Statistical analyses were performed separately for female and male groups.

**Stress Regimen**			**Female**			
**Stress**	**Recovery**	**Region**	**wt**	**Epac1**^**-/-**^	**Epac2**^**-/-**^	**Epac1/2**^**-/-**^
		DG	4.42±0.96 F(3,8) = 24.25, p = 0.0002	19.71±1.54 F(3,8) = 2.94, p = 0.10	13.68±2.86 F(3,8) = 0.93, p = 0.47	6.25±0.86 F(3,8) = 14.44, p = 0.0014
No	No	CA1	5.96±1.17 F(3,8) = 18.42, p = 0.0006	24.84±1.73 F(3,8) = 1.83,p = 0.22	21.42±1.23 F(3,8) = 1.03, p = 0.43	7.10±0.94 F(3,8) = 99.45, p<0.0001
		CA3	3.09±0.88 F(3,8) = 13.84, p = 0.0016	9.89±2.83 F(3,8) = 0.35, p = 0.79	9.32±1.46 F(3,8) = 3.69, p = 0.06	5.15±0.77 F(3,8) = 18.08, p = 0.0006
		DG	15.69±3.16^aaa^	15.22±1.21	12.40±4.87	5.75±2.02
30min	0h	CA1	23.07±2.38^aaa^	21.51±2.79	19.57±4.37	6.29±2.65
		CA3	12.96±3.90^aa^	9.78±1.90	11.61±1.87	3.73±1.93
		DG	7.25±0.27^bb^	16.39±2.33	16.30±4.59	5.09±0.70
30min	30min	CA1	13.40±5.43^b^	25.02±0.25	22.15±1.96	5.23±0.81
		CA3	4.79±0.26^bb^	8.84±2.40	10.28±0.53	4.08±0.09
		DG	7.57±0.81^bb^	15.87±2.65	16.67±1.39	21.38±4.00^aa^^,^^bb^^,^^cc^
30min	2h	CA1	8.44±0.69^bb^	22.26±3.18	23.01±0.62	24.78±1.38^aaaa^^,^^bbbb^^,^^cccc^
		CA3	5.49±0.71^bb^	10.58±0.13	13.08±1.62	13.30±2.37^aa^^,^^bbb^^,^^cc^
			**Male**			
**Stress**	**Recovery**	**Region**	**wt**	**Epac1**^**-/-**^	**Epac2**^**-/-**^	**Epac1/2**^**-/-**^
		DG	17.40±4.42 F(3,10) = 5.24, p = 0.02	10.75±1.68 F(3,8) = 1.73, p = 0.24	13.75±4.74 F(3,9) = 0.34, p = 0.80	9.97±6.07 F(3,8) = 1.58, p = 0.27
No	No	CA1	22.00±6.04 F(3,10) = 4.66, p = 0.03	15.79±3.21 F(3,8) = 4.77, p = 0.03	21.23±6.31 F(3,9) = 0.20, p = 0.89	23.82±7.32 F(3,8) = 1.55, p = 0.28
		CA3	12.08±3.49 F(3,10) = 2.60, p = 0.11	7.04±1.64 F(3,8) = 2.40, p = 0.14	8.63±5.07 F(3,9) = 0.30, p = 0.83	12.94±5.27 F(3,8) = 3.42, p = 0.07
		DG	18.54±3.00	19.31±9.09	15.90±1.84	17.70±6.86
30min	0h	CA1	23.61±1.89	26.81±4.93^a^	22.90±2.64	25.11±0.80
		CA3	13.49±4.01	12.50±4.99	10.17±0.67	15.77±0.23
		DG	13.26±7.72	12.06±2.19	12.91±4.52	14.28±5.61
30min	30min	CA1	19.80±9.90	21.78±1.36	20.40±5.35	18.76±5.91
		CA3	9.85±7.39	7.66±1.22	8.17±3.26	7.59±1.36
		DG	6.86±2.56^b^	11.50±4.37	14.00±3.52	8.45±1.37
30min	2h	CA1	10.45±1.82^b^	21.93±3.83	22.20±0.66	13.49±1.98
		CA3	5.07±2.69	9.46±0.92	8.09±1.35	4.39±1.32

^a^p<0.05

^aa^p<0.01

^aaa^p<0.001 and

^aaaa^p<0.0001: unstressed (No) mice compared to mice subjected to 30min stress with recovery (0h, 30min or 2h), same genotype, sex and brain region.

^b^p<0.05

^bb^p<0.01

^bbb^p<0.001 and

^bbbb^p<0.0001: mice subjected to 30min stress, no recovery compared to mice subjected to 30min stress with recovery (30min or 2h), same genotype, sex and brain region.

^cc^p<0.01 and

^cccc^p<0.0001: mice subjected to 30min stress, with 30min recovery compared to mice subjected to 30min stress with 2h recovery, same genotype, sex and brain region. n = 3 mice per group. For each mouse, 3 sections of the hippocampus where quantified for GR staining. F-statistics (F(Dfn, DFd)) for each comparison is indicated next to the mean ±SEM values in the unstressed (No) groups.

In female wt mice, nuclear GR staining increased immediately after restraint stress and decreased again after 30min and 2h of recovery in all regions (Fig 3A–3C and 3G and Table 1). Interestingly, female Epac1/2^-/-^ mice deviated clearly from wt females with a delayed GR response (Fig 3A–3C and 3G and Table 1), and furthermore, female Epac1^-/-^ and Epac2^-/-^ mice differed from wt with a more intense GR staining in the cell soma across all regions in the resting state (Fig 3A–3C and S3 Table), and upon stress followed by 30min and 2h recovery (Fig 3A–3C), resulting in a flattened response in these genotypes (Table 1). The transient increase in nuclear staining observed in female wt mice was not observed in wt male mice (Table 1). This finding in male mice is partly in conflict with previous studies on mice that report nuclear translocation of GR in response to stress [29]. However, of note is that minor effects of GR translocation upon stress have been reported earlier in male mice [57, 58]. GR nuclear staining was higher in unstressed wt male than in unstressed wt female mice (S3 Table). We have not found published studies comparing GR cellular distribution in resting female and male mice, and it is at present unclear whether this observed sex difference reflects the natural state, or whether the control male mice were unintentionally mildly stressed during handling. Overall, the differences between wt mice and Epac1^-/-^, Epac2^-/-^ and Epac1/2^-/-^ mice were less profound in the male group than in the female group (Fig 3 and Table 1). This was particularly evident in the CA1 region, which expresses high levels of GR [59]. According to the literature, the GR dependent responses in hippocampus after stress is mainly caused by redistribution of GR and posttranslational modifications, and not by changes in GR expression levels [29–31]. In line with this, the levels of GR mRNA were not changed in response to stress in either genotype or sex (S4 Table). Moreover, the only difference in GR mRNA expression among genotypes that were observed was between female Epac1^-/-^ and Epac2^-/-^ (S7 Fig). The potential physiological significance of this difference is at present unclear.

### The expression of hippocampal miR-124 is repressed in female Epac1/2Epac knockout models

**Fig 4:**
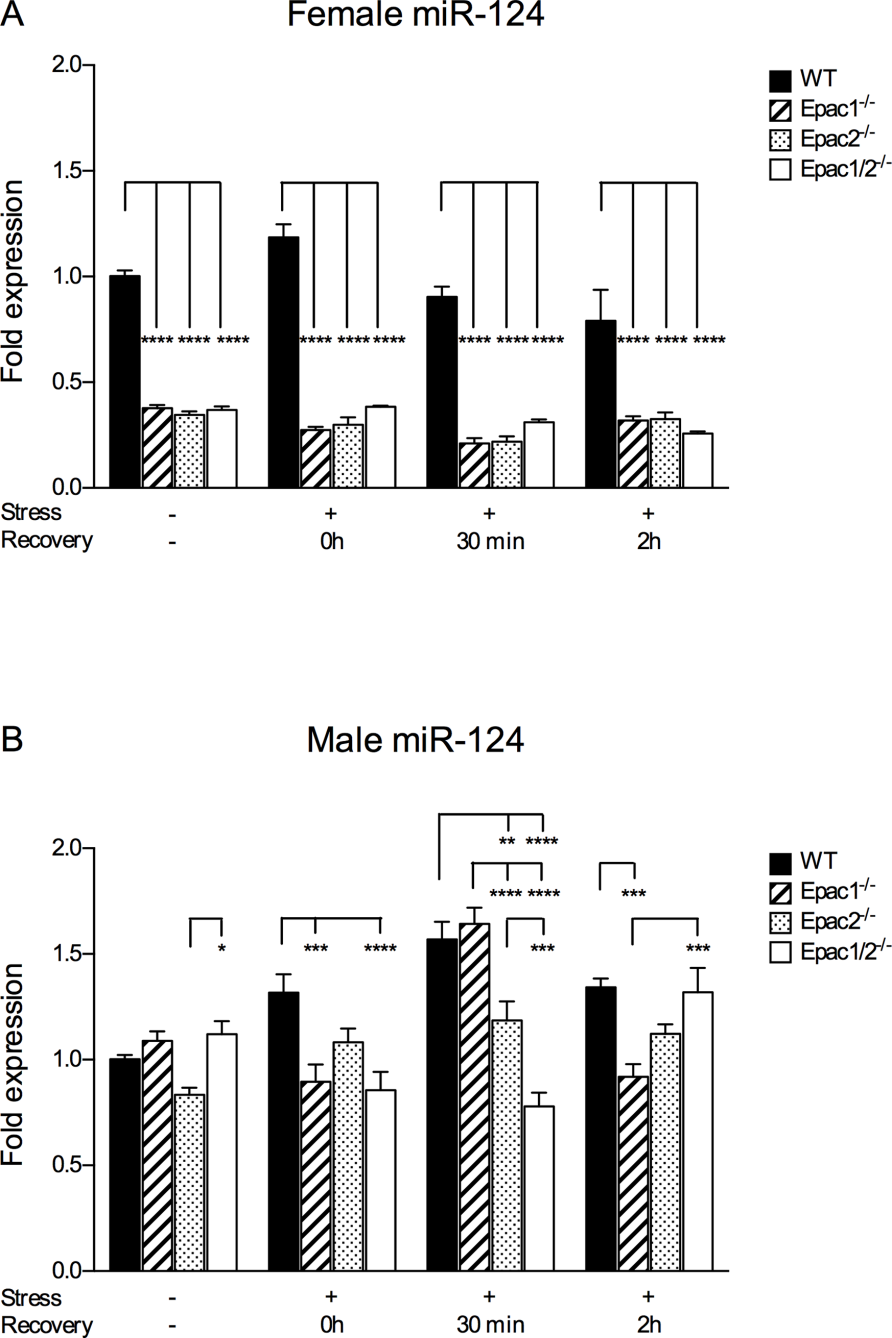
MiR-124 expression is decreased in hippocampus of female mice lacking Epac. qPCR analyses of miR-124 were performed on cDNA prepared from hippocampus from female **(A)** and male **(B)** wt, Epac1^-/-^, Epac2^-/-^ and Epac1/2^-/-^ mice kept at standard housing conditions (-) or exposed to 30min restraint stress. The hippocampus was dissected out after the mice had been culled immediately after the stressor (0h), or after recovery from the stress for 30min or 2h. The qPCR values were normalized to the expression of the reference small nuclear RNAs Snord66, Snord47 and Rnu6, and shown as average of relative fold change ±SEM of three independent experiments performed in triplicates with cDNA prepared from mRNA pooled from the hippocampus from 4–7 mice per group. Two-way ANOVA with Tukey’s adjustment for multiple comparisons was used to determine differences between genotypes in each treatment group Statistical analyses were performed separately for the female and male groups. *p≤0.05, **p<0.01, ***p<0.001 and ****p<0.0001. F-statistics (F(Dfn, DFd)) for the female group: Interaction: F(9, 122) = 4.359, p<0.0001 and the male group: Interaction: F(9, 128) = 12.90, p<0.0001.

The brain enriched microRNA miR-124 has previously been implicated in acute hippocampal stress responses [60], and in Epac1/2Epac dependent signaling [23, 61]. We therefore determined whether deletion of Epac1/2Epac affected hippocampal miR-124 expression in our experimental set up. Interestingly, hippocampal miR-124 expression was consistently reduced in female Epac1^-/-^, Epac2^-/-^ and Epac1/2^-/-^ mice compared to wt mice, both under resting conditions and after restraint (Fig 4A). Decreased miR-124 expression was also evident as a consequence of deleting Epac1 and/or Epac2 in the male group, but to a lesser degree and less consistently than in the female group (Fig 4B). Differences were also observed between the male Epac knockout models (Fig 4B). In the wt female group, miR-124 expression was slightly increased immediately after 30min of stress, and declined back to basal levels upon recovery (S5 Table). The stress regimen did not affect miR-124 levels in female Epac1^-/-^, Epac2^-/-^ and Epac1/2^-/-^ mice. Mir-124 expression also increased in wt males in response to stress (S5 Table), whereas the male Epac knockout models exhibited a variable expression pattern of miR-124 (S5 Table). The expression of Ngfi-A is induced in mouse hippocampus in response to stress [62] and because this factor has also been suggested as a target for miR-124 in Epac1/2^-/-^ mice [23] we determined the expression of Ngfi-A (S8 Fig). Ngfi-A expression was induced in response to stress in all female genotypes, and declined upon recovery (S6 Table). The trend was similar in the male group, but less significant (S6 Table). Ngfi-A expression was affected by genotype to some extent in some of the treatment groups, but no consistent pattern was observed (S8 Fig).

## Discussion

Signaling cascades initiated by cAMP are important for hippocampal functions, and also at all levels of the HPA axis where cAMP is fundamental for controlling hormonal output and feedback responses. PKA conveys cAMP induced signaling at these sites, but in more recent years it has become evident that some of these functions are Epac-dependent [11]. In the present study, we identified phenotypic characteristics that imply the involvement of Epac in hippocampal responses to acute stress. Intriguingly, some of these features, such as delayed GR translocation and consistent reduced miR124 expression were detected only in female mice. Additionally, increased mRNA expression of Epac1 and Epac2 in hippocampus upon stress exposure was specific for female mice, indicating sex-dependent differences in Epac signaling in connection to stress. This is the first report to describe acute stress related phenotypes in Epac knockout models. However, a previous study demonstrates abnormal behavior of Epac1/2^-/-^ mice in social stressful situations [23]. Furthermore, a study based on microarray hybridization reported decreased Epac2 expression in male hippocampus after exposure to a combination of chronic and acute stressors [63]. Although these studies are not directly comparable, together they point to functional roles for Epac in stress responses in the mouse brain.

Both Epac1 and Epac2 are expressed in the mouse hippocampus, with Epac2 at significantly higher levels than Epac1 in adult mice [10, 17]. Examination of different Epac knockout models demonstrates a variety of defects in hippocampal functions. For instance, axon elongation and polarization are affected in hippocampal neurons isolated from Epac1^-/-^ mice [22], and genetic ablation of Epac1 causes reduced transmitter release at mossy fiber CA3 synapses and impaired long term plasticity [19], as well as reduced N-methyl-D-aspartate (NMDA) receptor dependent long-term depression and behavioral flexibility in spatial learning and memory [20]. A recent study also describes impaired neurogenesis and increased anxiety and depression in the absence of Epac2, but not Epac1 [21]. In a different model, deletion of both Epac1 and Epac2 was required to provoke a phenotype of deficits in spatial learning and social interactions [23]. Thus, these studies indicate distinct functional roles for Epac1 and Epac2 in hippocampal physiology. The data presented in the current study also support this idea, since female Epac1/2^-/-^ mice exhibit a GR translocation phenotype that deviates not only from wt mice, but also from Epac1^-/-^ and Epac2^-/-^ mice. Moreover, the concept that Epac1 and Epac2 have distinct functions in the hippocampus is strengthened by the observation that intra-hippocampal injection of the Epac activator 8-pCPT-2’O-Me-cAMP, known to preferentially activate Epac1 [64], facilitates memory retrieval, whereas injection of siRNA targeting Epac2 impairs fear memory retrieval [17].

GR is highly expressed in the hippocampus, and several studies demonstrate that irregular GR expression in the forebrain alters behavior in mice. Analyses of knockout models have revealed distinct roles for GR in the manifestation of depressive- and anxiety-like symptoms, demonstrating that it is essential to control GR transcriptional activity in stressful situations [65]. GR and cAMP dependent signaling are integrated in the hippocampus [37] as well as in the HPA axis [66]. Our result demonstrating that stress-induced nuclear accumulation of GR is significantly delayed in Epac1/2^-/-^ female mice provides new insights into how these pathways are interrelated, and also suggest sex-dependent differences. Sex specific differences in GR nuclear translocation have previously been reported in adolescent rats exposed to the forced swim test for 5 min in combination with chronic adolescent stress [57].

In recent years, several studies indicate roles for Epac, especially Epac1, in nuclear functions. Epac1 is localized to the nuclear pore in several cell types [67, 68], and Epac1 is also part of a nuclear complex with a PKA anchoring protein in cardiomyocytes [69]. Epac2B, which is the Epac2 isoform with highest similarity to Epac1, is also enriched in nuclear fractions [70]. On the other hand, it has been postulated that the N-terminal cAMP-binding motif in Epac2A (not found in Epac2B) blocks the nuclear pore localization signal [68]. In line with this, Epac2A is localized to the plasma membrane and to membrane structures in the cytoplasm [25, 71]. The underlying mechanisms causing altered GR translocation in female Epac1/2^-/-^ mice are yet to be determined. However, in this regard it is interesting to note that GR localization is affected by cAMP, as activation of this pathway by β2-adrenergic receptor agonists causes induced GR nuclear translocation in Epac1 expressing lung fibroblasts and vascular smooth muscle cells [72–74]. Moreover, Epac have also been implicated in the regulation of nuclear translocation of other proteins. In osteoclasts, activation of Epac modulates translocation of the transcription factor NFkB (nuclear factor kappa-light-chain-enhancer of activated B cells) [75], and in various other cell types, Epac and PKA together regulate the nuclear/cytoplasmic trafficking of the DNA-dependent protein kinase (DNA-PK) [76].

The GR expression level is regulated by the brain enriched microRNA miR-124. The 3’ untranslated region of *Nr3c1* contains a target site for miR-124, and *in vitro* experiments demonstrate that miR-124 leads to decreased GR expression [38]. Moreover, in both mice [77] and rats [78], increased miR-124 expression is correlated with decreased GR levels and also with GC sensitivity. MiR-124 has not been analyzed in female Epac knockout models before, but forebrain specific male Epac1/2^-/-^ mice exhibit increased miR-124 expression (no effects on miR-124 levels were observed in male Epac1^-/-^ or Epac2^-/-^ mice) [23]. Further linking Epac signaling and miR-124 expression is the finding that the Epac1 activator 8-pCPT-2’O-Me-cAMP causes reduced miR-124 expression in hippocampal neurons isolated from male rats [61]. We observed decreased expression of miR-124 in female mice deleted for Epac1, Epac2 or both factors compared to wt mice. This effect was independent of stress exposure, and clearly indicates that the mechanisms controlling miR-124 expression are at least partly defective in female mice lacking Epac. In support of a sex specific mode of miR-124 expression are two reports demonstrating differently expressed miR-124 expression in the developing ovary compared to the male anlagen [79], and in adipose tissue of obese male and female pigs [80]. Based on the established role of miR-124 in the control of GR expression [38, 77–78], it might have been expected that the decreased miR-124 expression observed in female Epac1^-/-^, Epac2^-/-^ and Epac1/2^-/-^ would result in increased GR mRNA expression. However, we did not observe elevated GR mRNA expression, and it is still unknown whether a direct mechanistic link exists between the delayed GR response and the decreased miR-124 expression that we observed in female Epac1/2^-/-^ mice.

The transcription factor Ngfi-A is induced by cAMP signaling and increases transcription from *Nr3c1* in hippocampal neurons [36], and it has also been suggested that expression of Ngfi-A is regulated by miR-124 [23, 81]. Interestingly, it was previously postulated that Ngfi-A expression is suppressed in male hippocampus as a consequence of reduced miR-124 expression, again caused by deletion of both Epac1 and Epac2 (no effects were observed in Epac1^-/-^ and Epac2^-/-^ mice) [23]. In contrast, we did not observe decreased Ngfi-A mRNA expression in male Epac1/2^-/-^ mice under resting conditions, but after stress and 2h recovery in males. Similarly, in the female group, Ngfi-A expression was decreased in all female Epac knockout models after 2h of recovery from stress. However, the mechanistic link suggested to exist between Epac, miR-124 and Ngfi-A by Yang and colleagues [23] in male mice was not confirmed by our results, although we present data that in female mice, deletion of Epac leads to a substantial decrease in miR-124 expression and delay in GR translocation after stress. At present, we cannot explain the inconsistency with regard to miR-124 or Ngfi-A expression in unstressed Epac1/2^-/-^ male mice, although it should be noted that different targeting strategies were used in the current study and the study presented in [23]. Whereas we employed models with global deletion of Epac1 and/or all isoforms of Epac2, Yang *et*. *al* utilized a model in which the Epac1 gene is specifically targeted in the forebrain in combination with global targeting of the 5’ part of the Epac2 gene [23]. (*I*.*e*. the transgene cassette was inserted in the region encoding exons 1–4 allowing normal RT-PCR amplification of the region between exons 4 and 5 [23], and possibly translation of Epac2B and Epac2C [15]). However, despite the discrepancies between the present study and [23], both studies suggest that Epac and miR-124 dependent signaling integrate, and further indicate that the regulation of these pathways might differ between male and female.

The neuroendocrine systems of men and women respond differently to stressors, causing sex differences in disease susceptibility, with women being more vulnerable to stress induced anxiety and depression. Sex differences have been confirmed at the molecular level and involve multiple pathways, including GC and GR signaling, although the understanding of the underlying biochemical pathways is still incomplete [82]. Taken together, the results presented in this report implicate sex specific differences in Epac signaling cascades in the mouse hippocampus. The distinct differences between males and females establish the Epac knockout models as valuable genetic tools to provide molecular insights into why the incidences of stress related diseases vary among sexes. The disclosure of disease-related phenotypes in Epac1/2^-/-^ mice, and the association with insulin secretion [25, 83] and diseases such as Alzheimer’s disease [84] and depression [85], has prompted considerable efforts to develop compounds that target either Epac1 or Epac2 without affecting PKA signaling [8, 64, 86]. These agonists hold promise for insights into isoform-specific functions, and for selective pharmacological targeting of Epac (reviewed in [87]).

## Supporting information

S1 Fig Mice lacking Epac show normal dexamethasone feedback response. Female (A) and male (B) wt, Epac1^-/-^, Epac2^-/-^ and Epac1/2^-/-^ mice received an intraperitoneal (IP) injection of dexamethasone or buffered saline. Six hours after the injection, the mice were culled and blood was collected and analyzed for serum corticosterone levels. Data are presented as a percent reduction in corticosterone after dexamethasone injection relative to the corresponding control (IP with buffered saline). One-way ANOVA revealed no significant differences between the groups of mice. n = 4 mice per group. F-statistics (F(Dfn, DFd)) for the female group: F(3, 12) = 1.301, p = 0.3191 and the male group: F(3, 12) = 1.886, p = 0.1857.
(PPTX)

S2 Fig Female and male mice deleted for Epac1 and/or Epac2 show normal hippocampal morphology. Coronal brain tissue sections from female (a) and male (b) wt, Epac1^-/-^, Epac2^-/-^, and Epac1/2^-/-^ mice were stained with H&E and visualized with a Leica DMLB light microscope. The images were acquired with a Leica DC 300 camera (Leica Microsystems AG). Nuclear staining of neuronal cell bodies is observed in the stratum pyramidale (SPy) for CA1 and CA3 pyramidal neurons, and the stratum granulosum (SG) for the DG granular neurons. Sparse nuclear staining can also be observed in the other layers of the hippocampus (moleculare (SM) oriens (SO) and radiatum (SR) layers). The 500μm scale bar in the lower right panel applies to all images shown in the figure.
(PPTX)

S3 Fig Epac1 and Epac2 levels are not increased in a compensatory manner in the hippocampus of the opposite knockout model. The mRNA expression of Epac1 and Epac2 was examined by qPCR in hippocampus tissue of Epac2^-/-^ (A) and Epac1^-/-^ (B) mice and compared to wt mice. The qPCR values were normalized to the expression of the housekeeping genes Sdha and Ppib, and are shown as average of fold expression ±SEM of four independent experiments performed in triplicates (n = 7–11). One-way ANOVA was used for statistical analysis. No significant differences were found. A) F-statistics F(DFn, DFd); F(3, 35) = 0.2710, p = 0.8459 B) F-statistics F(DFn, DFd); F(3, 30) = 0.0110, p = 0.9984
(PPTX)

S4 Fig GR immunofluorescence of hippocampal cell soma in the CA1 stratum pyramidale, the DG stratum granulosum (SG) and the CA3 stratum pyramidale neuronal layers in female mice. Female wt, Epac1/2^-/-^, Epac1^-/-^ or Epac2^-/-^ mice were kept at standard housing conditions (unstressed) or exposed to 30min of restraint stress, and either culled immediately after the stressor (0h recovery), or after recovery from the stress for 30min or 2h. Paraffin-embedded coronal brain sections (15μm) were stained with a GR-specific antibody and thereafter visualized under a) a 60X objective of the Nikon Te 2000-e microscope with a TRITC fluorescent light filter, and captured with a Nikon Digital Sight DS-U1 camera or b-d) a Cy3 fluorescent light filter at 590nm at 10X magnification with an Axioplan 2 Imaging-e immunofluorescence microscope, and images captured with a Zeiss Axiocam HR digital camera. GR immunofluorescence was also observed outside the pyramidale layer; in the Stratum oriens (SO), and radiatum (SR) layers (b) and outside the Stratum granulosum layer; in the Stratum Moleculare (SM) layer (c). a) The 10μm scale bar in the lower right panel applies to all images shown in the figure. b-d) The 100μm scale bar in the lower right panel applies to all images shown in the figures. CA: Cornu Ammonis; Spy: Stratum Pyramidale; SR: Stratum Radiatum; SO: Stratum Oriens; SG: Stratum Granulosum; SM: Stratum Moleculare.
(PPTX)

S5 Fig GR immunofluorescence of hippocampal cell soma in the CA1 stratum pyramidale, the DG stratum granulosum (SG) and the CA3 stratum pyramidale neuronal layers in male mice. Male wt, Epac1/2^-/-^, Epac1^-/-^ or Epac2^-/-^ mice were kept at standard housing conditions (unstressed) or exposed to 30min of restraint stress, and either culled immediately after the stressor (0h recovery), or after recovery from the stress for 30min or 2h. Paraffin-embedded coronal brain sections (15μm) were stained with a GR-specific antibody and thereafter visualized under a) a 60X objective of the Nikon Te 2000-e microscope with a TRITC fluorescent light filter, and captured with a Nikon Digital Sight DS-U1 camera or b-d) a Cy3 fluorescent light filter at 590nm at 10X magnification with an Axioplan 2 Imaging-e immunofluorescence microscope, and images captured with a Zeiss Axiocam HR digital camera. GR immunofluorescence was also observed outside the pyramidale layer; in the Stratum oriens (SO), and radiatum (SR) layers (b) and outside the Stratum granulosum layer; in the Stratum Moleculare (SM) layer (c). a) The 10μm scale bar in the lower right panel applies to all images shown in the figure. b-d) The 100μm scale bar in the lower right panel applies to all images shown in the figures. CA: Cornu Ammonis; Spy: Stratum Pyramidale; SR: Stratum Radiatum; SO: Stratum Oriens; SG: Stratum Granulosum; SM: Stratum Moleculare.
(PPTX)

S6 Fig GR and DAPI staining of the CA1 stratum pyramidale layer in wt mice. Wt, Epac1/2^-/-^, Epac1^-/-^ or Epac2^-/-^ mice were kept at standard housing conditions (unstressed) or exposed to 30min of restraint stress, and either culled immediately after the stressor (0h recovery), or after recovery from the stress for 30min or 2h. Paraffin-embedded coronal brain sections (15μm) were stained with a GR-specific antibody and thereafter visualized under a 60X objective of the Nikon Te 2000-e microscope with a TRITC fluorescent light filter, and captured with a Nikon Digital Sight DS-U1 camera. The 10μm scale bar in the lower right panel applies to all images shown in the figure. As shown in the figure, GR immunofluorescence staining was confined to the nuclear regions of the hippocampal cell soma.
(PPTX)

S7 Fig Expression of GR mRNA in wt, Epac1^-/-^, Epac2^-/-^ and Epac1/2^-/-^ mice following restraint stress. Female (**A**) and male (**B**) mice were kept at standard housing conditions (-) or exposed to 30min of restraint stress (+). The hippocampus was dissected out after the mice had been culled immediately after the stressor (0h), or after recovery from the stress for 30min or 2h, and mRNA prepared. qPCR analyses were performed to determine GR mRNA levels. The qPCR values were normalized to the expression of the housekeeping genes Sdha and Ppib, and are shown as average of relative fold change ±SEM of three independent experiments performed in triplicates (n = 7–9). Two-way ANOVA with Tukey’s adjustment for multiple comparisons was used for statistical analysis. F-statistics (F(Dfn, DFd)) for the female group: Interaction: F(9, 113) = 0.3331, p = 0.9623 and the male group: Interaction: F(9, 119) = 0.5079, p = 0.8664
(PPTX)

S8 Fig Expression of Ngfi-A mRNA in wt, Epac1^-/-^, Epac2^-/-^ and Epac1/2^-/-^ mice following restraint stress. Female (**A**) and male (**B**) mice were kept at standard housing conditions (-) or exposed to 30min of restraint stress (+). The hippocampus was dissected out after the mice had been culled with CO_2_ immediately after the stressor (0h), or after recovery from the stress for 30min or 2h, and mRNA prepared. qPCR analyses were performed to determine Ngfi-A mRNA levels. The qPCR values were normalized to the expression of the housekeeping genes Sdha and Ppib, and are shown as average of relative fold change, ± SEM of three independent experiments performed in triplicates (n = 7–9). Two-way ANOVA with Tukey’s adjustment for multiple comparisons was used for statistical analysis. F-statistics (F(Dfn, DFd)) for the female group: Interaction: F(9, 128) = 6.755, p<0.0001 and the male group: Interaction: F(9, 118) = 3.383, p = 0.0010.
(PPTX)

S1 Table List of primers used in qPCR experiments. (PPTX)

S2 Table Comparison of serum corticosterone levels in unstressed *vs*. stressed mice. Based on the results shown in Fig 1, serum corticosterone levels of unstressed (-) and stressed (0h, 30min and 2h) mice (shown as mean ± SD) were compared and significance determined by Two-way ANOVA with Tukey's adjustment for multiple comparisons. Statistical analyses were performed separately for the female and male groups. ^aa^p<0.01, ^aaa^p<0.001 and ^aaaa^p<0.0001 unstressed mice (-) compared to mice subjected to 30min stress with recovery (0h, 30min or 2h), same genotype and sex. ^b^p<0.5, ^bbb^p<0.001 and ^bbbb^p<0.0001 mice subjected to 30min stress, no recovery compared to mice subjected to 30min stress with recovery (30min or 2h). ^c^p<0.5 and ^ccc^p<0.001 mice subjected to 30min stress with 30min recovery compared to mice subjected to 30min stress with 2h recovery, same genotype and sex. n = 7–9 mice per group. F-statistics (F(Dfn, DFd)) for the female group: Interaction: F(9,102) = 3.713, p = 0.0005 and the male group: Interaction: F(9,108) = 1.759, p = 0.0846.
(PPTX)

S3 Table Comparison of GR immunofluorescence in unstressed female *vs*. male mice. Based on the results shown in Fig 3, GR staining in unstressed female and male mice (all genotypes, presented as average ±SD gray values) were compared and significance determined by One-way ANOVA with Tukey`s adjustment for multiple comparisons. Statistical analyses were performed separately for each region. ^a^p≤0.05, ^aa^p≤0.01,^aaa^p<0.001 and ^aaaa^p≤0.0001 unstressed (-) female wt mice compared to unstressed female Epac1^-/-^, Epac2^-/-^, Epac1/2^-/-^ mice and male mice (all genotypes), same region, ^b^p≤0.05, ^bb^p≤0.01,^bbb^p<0.001 and ^bbbb^p≤0.0001 unstressed female Epac1^-/-^ mice compared to unstressed female Epac2^-/-^, Epac1/2^-/-^ mice and male mice (all genotypes), same region. ^ccc^p<0.001 and ^cccc^p≤0.0001 unstressed female Epac2^-/-^ mice compared to unstressed female Epac1/2^-/-^ mice and male mice (all genotypes), same region. ^dd^p≤0.01, ^ddd^p<0.001 and ^dddd^p≤0.0001 unstressed female Epac1/2^-/-^ mice compared to male mice (all genotypes), same region. ^e^p≤0.05, ^ee^p≤0.01 and ^eee^p<0.001 unstressed male wt mice compared to unstressed male Epac1^-/-^, Epac2^-/-^ and Epac1/2^-/-^ mice, same region. n = 3 mice per group, and for each mouse, 3 sections of the hippocampus where quantified for GR staining. F-statistics (F(DFn, DFd)) DG; Interaction: F(7, 64) = 20.88, p<0.0001, CA1; Interaction: F(7, 64) = 25.46, p<0.0001 and CA3; Interaction: F(7, 64) = 8.804, p<0.0001.
(PPTX)

S4 Table Comparison of GR mRNA levels in unstressed *vs*. stressed mice. Based on the results shown in S7 Fig, GR mRNA levels in unstressed (-) and stressed (0h, 30min and 2h) mice were compared and significance determined by Two-way ANOVA The data is presented as average of relative fold change ± SEM of three independent experiments performed in triplicates (n = 7–9). Statistical analyses were performed separately for the female and male groups. No significant differences were found. F-statistics (F(Dfn, DFd)) for the female group: Interaction: F(9, 113) = 0.3331, p = 0.9623 and the male group: Interaction: F(9, 119) = 0.5079, p = 0.8664.
(PPTX)

S5 Table Comparison of miR-124 levels in unstressed *vs*. stressed mice. Based on the results shown in Fig 4, miR-124 levels in unstressed (-) and stressed (0h, 30min and 2h) mice were compared and significance determined by Two-way ANOVA with Tukey's adjustment for multiple comparisons. The data is presented as average of relative fold change ± SEM of three independent experiments performed in triplicates (n = 7–9). Statistical analyses were performed separately for the female and male groups. ^a^p<0.05 ^aa^p<0.01 and ^aaaa^p<0.0001 unstressed mice (-) compared to mice subjected to 30min stress with recovery (0h, 30min or 2h), same genotype and sex. ^bbbb^p<0.0001 mice subjected to 30min stress, no recovery compared to mice subjected to 30min stress with recovery (30min or 2h), same genotype and sex. ^cccc^p<0.0001 mice subjected to 30min stress with 30min recovery compared to mice subjected to 30min stress and 2h recovery, same genotype and sex. F-statistics (F(Dfn, DFd)) for the female group: Interaction: F(9, 122) = 4.359, p<0.0001 and the male group: Interaction: F(9, 128) = 12.90, p<0.0001.
(PPTX)

S6 Table Comparison of Ngfi-A mRNA levels in unstressed *vs*. stressed mice. Based on the results shown in S8 Fig, Ngfi-A mRNA levels in unstressed (-) and stressed (0h, 30min and 2h) mice were compared and significance determined by Two-way ANOVA with Tukey's adjustment for multiple comparisons. The data is presented as average of relative fold change ± SEM of three independent experiments performed in triplicates (n = 7–9). Statistical analyses were performed separately for the female and male groups. ^a^p<0.05 ^aa^p<0.01, ^aaa^p<0.001 and ^aaaa^p<0.0001 unstressed mice (-) compared to mice subjected to 30min stress with recovery (0h, 30min or 2h), same genotype and sex. ^b^p<0.05, ^bb^p<0.01, ^bbb^p<0.001 and ^bbbb^p<0.0001 mice subjected to 30min stress, no recovery compared to mice subjected to 30min stress with recovery (30min or 2h), same genotype and sex. ^c^p<0.05, ^cc^p<0.01 and ^cccc^p<0.0001 mice subjected to 30min stress with 30min recovery compared to mice subjected to 30min stress with 2h recovery. F-statistics (F(Dfn, DFd)) for the female group: Interaction: F(9, 128) = 6.755, p<0.0001 and the male group: Interaction: F(9, 118) = 3.383, p = 0.0010.
(PPTX)
