# Supplementary figures and images for: Deletion of exchange proteins directly activated by cAMP (Epac) causes defects in hippocampal signaling in female mice
Source: PLoS One. 2018 Jul 26;13(7):e0200935. doi: 10.1371/journal.pone.0200935 (PMC6062027; doi:10.1371/journal.pone.0200935)

## Slide 1
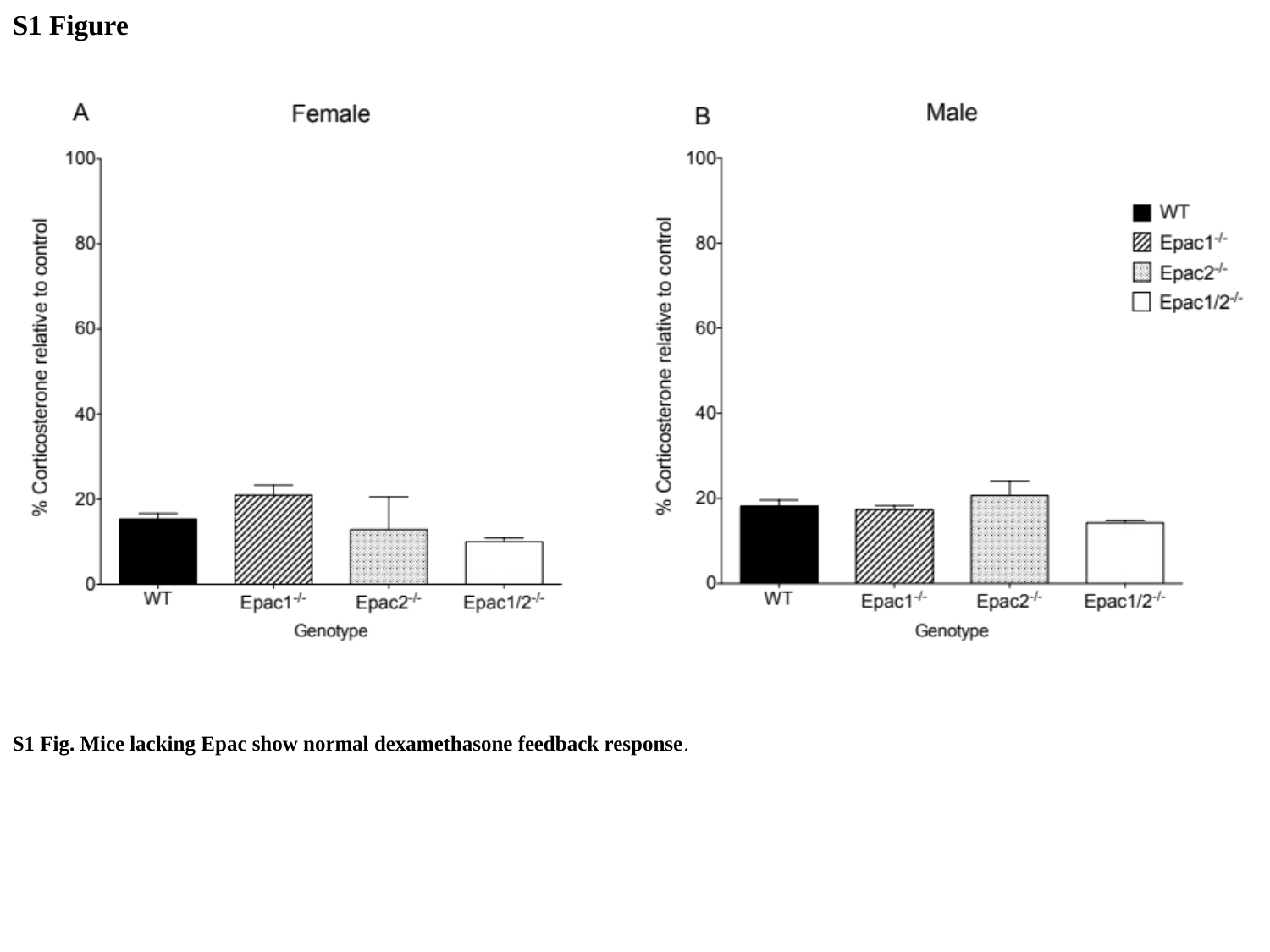

S1 Figure
S1 Fig. Mice lacking Epac show normal dexamethasone feedback response.

Supplement: S1 Fig — Female (A) and male (B) wt, Epac1-/-, Epac2-/- and Epac1/2-/- mice received an intraperitoneal (IP) injection of dexamethasone or buffered saline. Six hours after the injection, the mice were culled and blood was collected and analyzed for serum corticosterone levels. Data are presented as a percent reduction in corticosterone after dexamethasone injection relative to the corresponding control (IP with buffered saline). One-way ANOVA revealed no significant differences between the groups of mice. n = 4 mice per group. F-statistics (F(Dfn, DFd)) for the female group: F(3, 12) = 1.301, p = 0.3191 and the male group: F(3, 12) = 1.886, p = 0.1857. (PPTX) [file pone.0200935.s001.pptx]
