# Supplementary material for: Deletion of exchange proteins directly activated by cAMP (Epac) causes defects in hippocampal signaling in female mice
Source: PLoS One. 2018 Jul 26;13(7):e0200935. doi: 10.1371/journal.pone.0200935 (PMC6062027; doi:10.1371/journal.pone.0200935)
Supplement: S2 Fig — Coronal brain tissue sections from female (a) and male (b) wt, Epac1-/-, Epac2-/-, and Epac1/2-/- mice were stained with H&E and visualized with a Leica DMLB light microscope. The images were acquired with a Leica DC 300 camera (Leica Microsystems AG). Nuclear staining of neuronal cell bodies is observed in the stratum pyramidale (SPy) for CA1 and CA3 pyramidal neurons, and the stratum granulosum (SG) for the DG granular neurons. Sparse nuclear staining can also be observed in the other layers of the hippocampus (moleculare (SM) oriens (SO) and radiatum (SR) layers). The 500μm scale bar in the lower right panel applies to all images shown in the figure. (PPTX) [file pone.0200935.s002.pptx]

## Slide 1
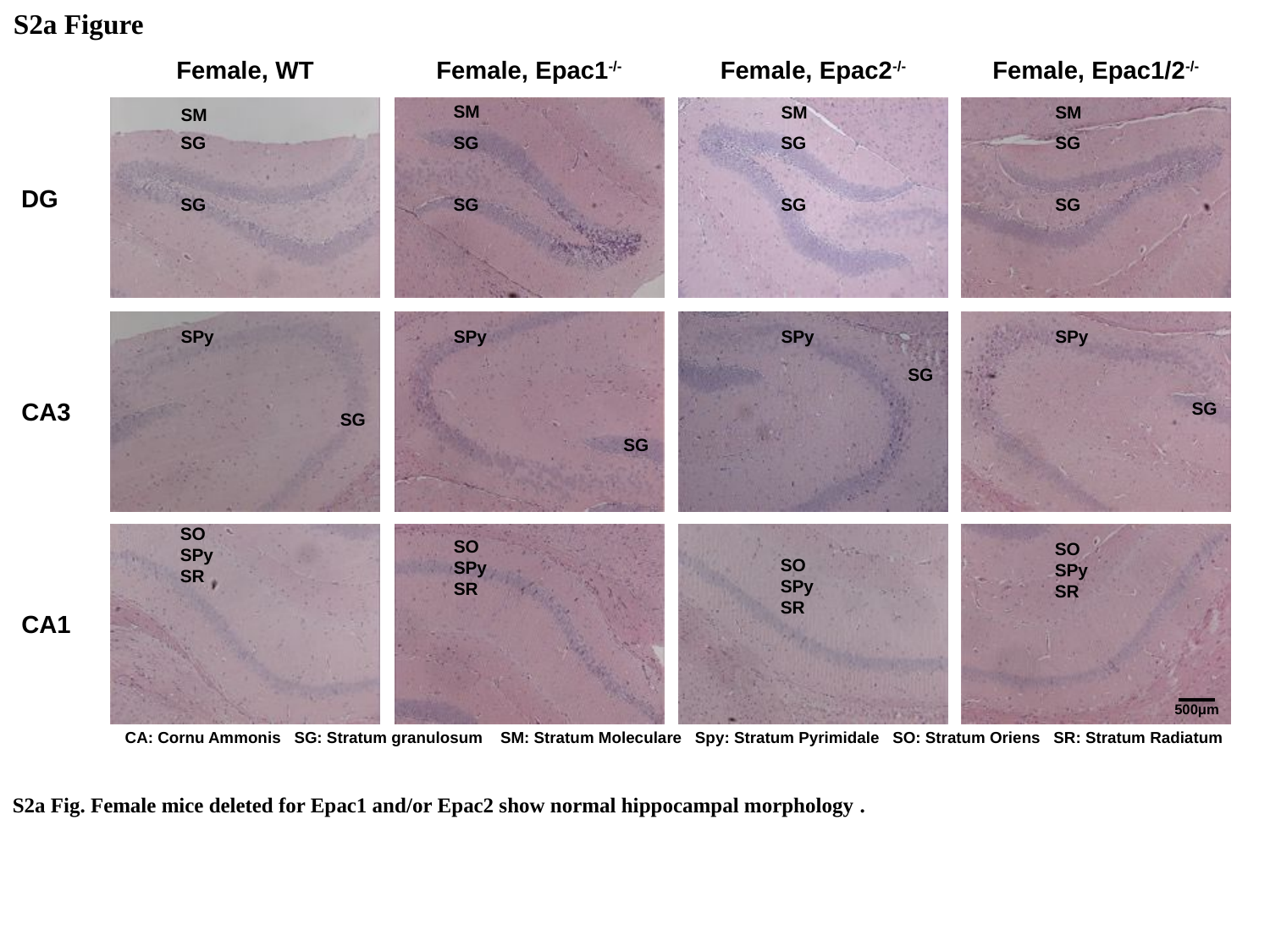

S2a Figure
Female, WT
Female, Epac1-/-
Female, Epac2-/-
Female, Epac1/2-/-
SM
SM
SM
SM
SG
SG
SG
SG
DG
SG
SG
SG
SG
SPy
SPy
SPy
SPy
SG
CA3
SG
SG
SG
SO
SPy
SR
SO
SPy
SR
SO
SPy
SR
SO
SPy
SR
CA1
500μm
CA: Cornu Ammonis SG: Stratum granulosum SM: Stratum Moleculare Spy: Stratum Pyrimidale SO: Stratum Oriens SR: Stratum Radiatum
S2a Fig. Female mice deleted for Epac1 and/or Epac2 show normal hippocampal morphology .

## Slide 2
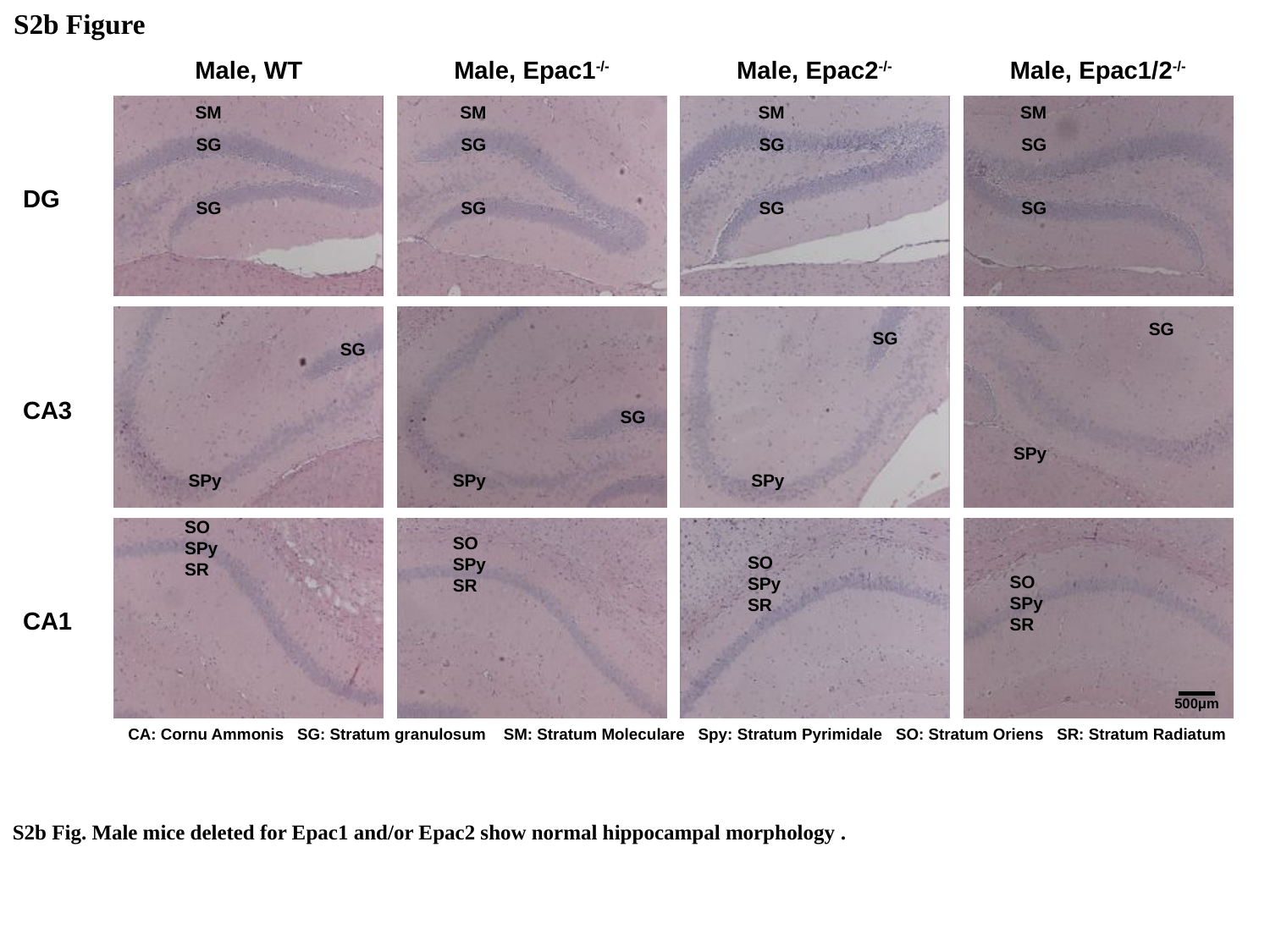

S2b Figure
Male, WT
Male, Epac1-/-
Male, Epac2-/-
Male, Epac1/2-/-
SM
SM
SM
SM
SG
SG
SG
SG
DG
SG
SG
SG
SG
SG
SG
SG
CA3
SG
SPy
SPy
SPy
SPy
SO
SPy
SR
SO
SPy
SR
SO
SPy
SR
SO
SPy
SR
CA1
500μm
CA: Cornu Ammonis SG: Stratum granulosum SM: Stratum Moleculare Spy: Stratum Pyrimidale SO: Stratum Oriens SR: Stratum Radiatum
S2b Fig. Male mice deleted for Epac1 and/or Epac2 show normal hippocampal morphology .
