# Supplementary material for: Deletion of exchange proteins directly activated by cAMP (Epac) causes defects in hippocampal signaling in female mice
Source: PLoS One. 2018 Jul 26;13(7):e0200935. doi: 10.1371/journal.pone.0200935 (PMC6062027; doi:10.1371/journal.pone.0200935)
Supplement: S3 Fig — The mRNA expression of Epac1 and Epac2 was examined by qPCR in hippocampus tissue of Epac2-/- (A) and Epac1-/- (B) mice and compared to wt mice. The qPCR values were normalized to the expression of the housekeeping genes Sdha and Ppib, and are shown as average of fold expression ±SEM of four independent experiments performed in triplicates (n = 7–11). One-way ANOVA was used for statistical analysis. No significant differences were found. A) F-statistics F(DFn, DFd); F(3, 35) = 0.2710, p = 0.8459 B) F-statistics F(DFn, DFd); F(3, 30) = 0.0110, p = 0.9984 (PPTX) [file pone.0200935.s003.pptx]

## Slide 1
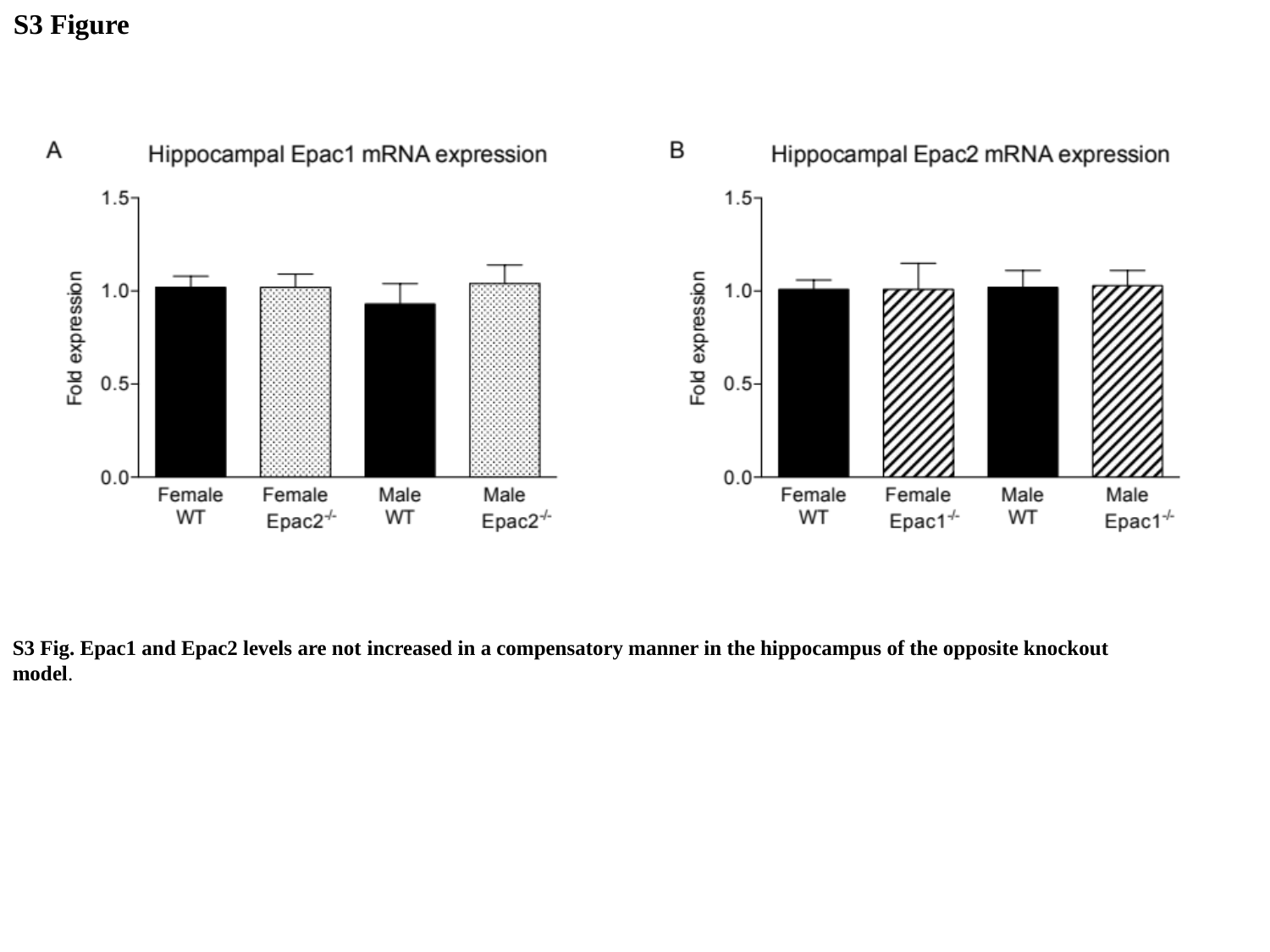

S3 Figure
S3 Fig. Epac1 and Epac2 levels are not increased in a compensatory manner in the hippocampus of the opposite knockout model.
