# Supplementary material for: Deletion of exchange proteins directly activated by cAMP (Epac) causes defects in hippocampal signaling in female mice
Source: PLoS One. 2018 Jul 26;13(7):e0200935. doi: 10.1371/journal.pone.0200935 (PMC6062027; doi:10.1371/journal.pone.0200935)
Supplement: S5 Fig — Male wt, Epac1/2-/-, Epac1-/- or Epac2-/- mice were kept at standard housing conditions (unstressed) or exposed to 30min of restraint stress, and either culled immediately after the stressor (0h recovery), or after recovery from the stress for 30min or 2h. Paraffin-embedded coronal brain sections (15μm) were stained with a GR-specific antibody and thereafter visualized under a) a 60X objective of the Nikon Te 2000-e microscope with a TRITC fluorescent light filter, and captured with a Nikon Digital Sight DS-U1 camera or b-d) a Cy3 fluorescent light filter at 590nm at 10X magnification with an Axioplan 2 Imaging-e immunofluorescence microscope, and images captured with a Zeiss Axiocam HR digital camera. GR immunofluorescence was also observed outside the pyramidale layer; in the Stratum oriens (SO), and radiatum (SR) layers (b) and outside the Stratum granulosum layer; in the Stratum Moleculare (SM) layer (c). a) The 10μm scale bar in the lower right panel applies to all images shown in the figure. b-d) The 100μm scale bar in the lower right panel applies to all images shown in the figures. CA: Cornu Ammonis; Spy: Stratum Pyramidale; SR: Stratum Radiatum; SO: Stratum Oriens; SG: Stratum Granulosum; SM: Stratum Moleculare. (PPTX) [file pone.0200935.s005.pptx]

## Slide 1
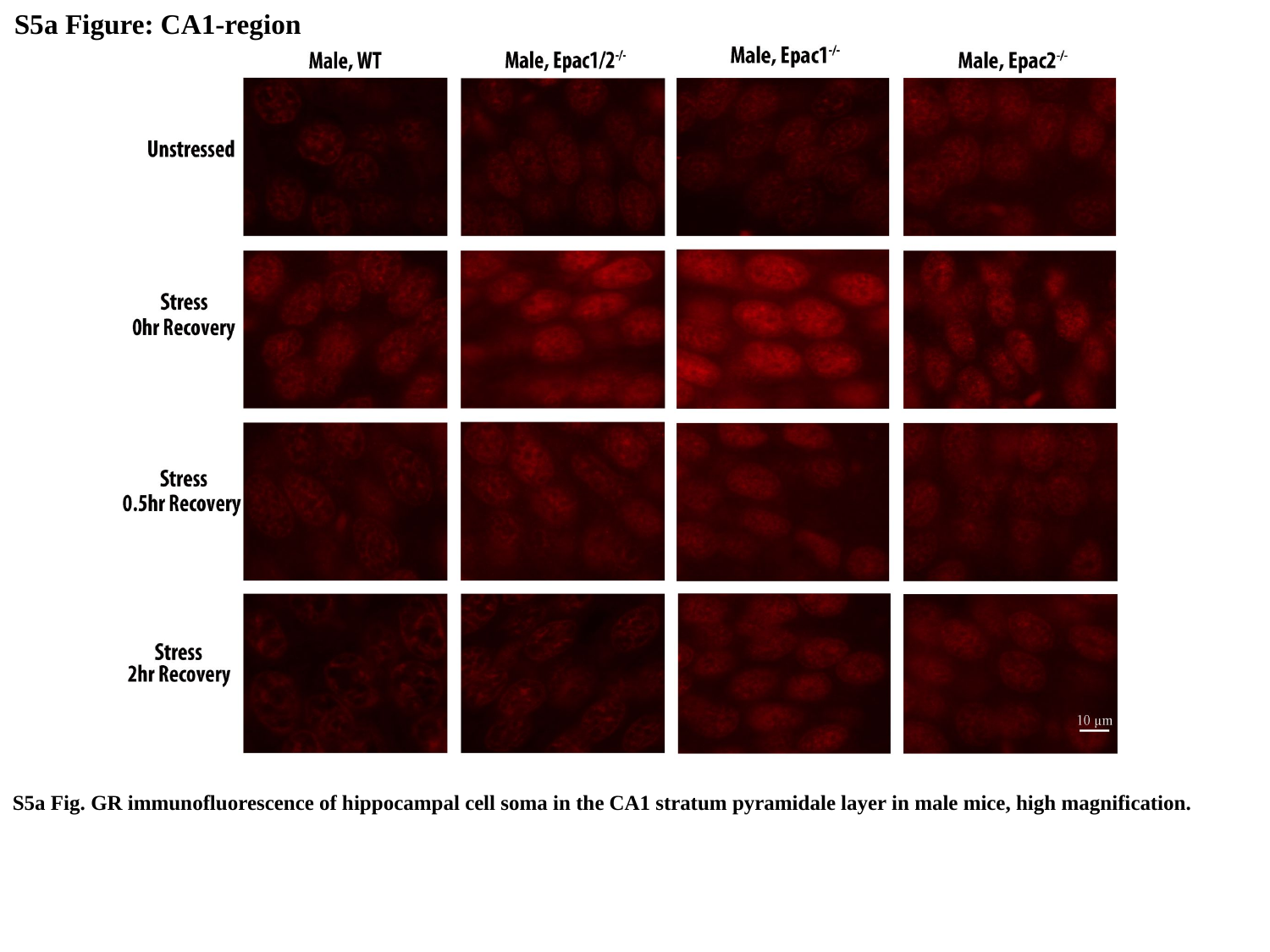

S5a Figure: CA1-region
S5a Fig. GR immunofluorescence of hippocampal cell soma in the CA1 stratum pyramidale layer in male mice, high magnification.

## Slide 2
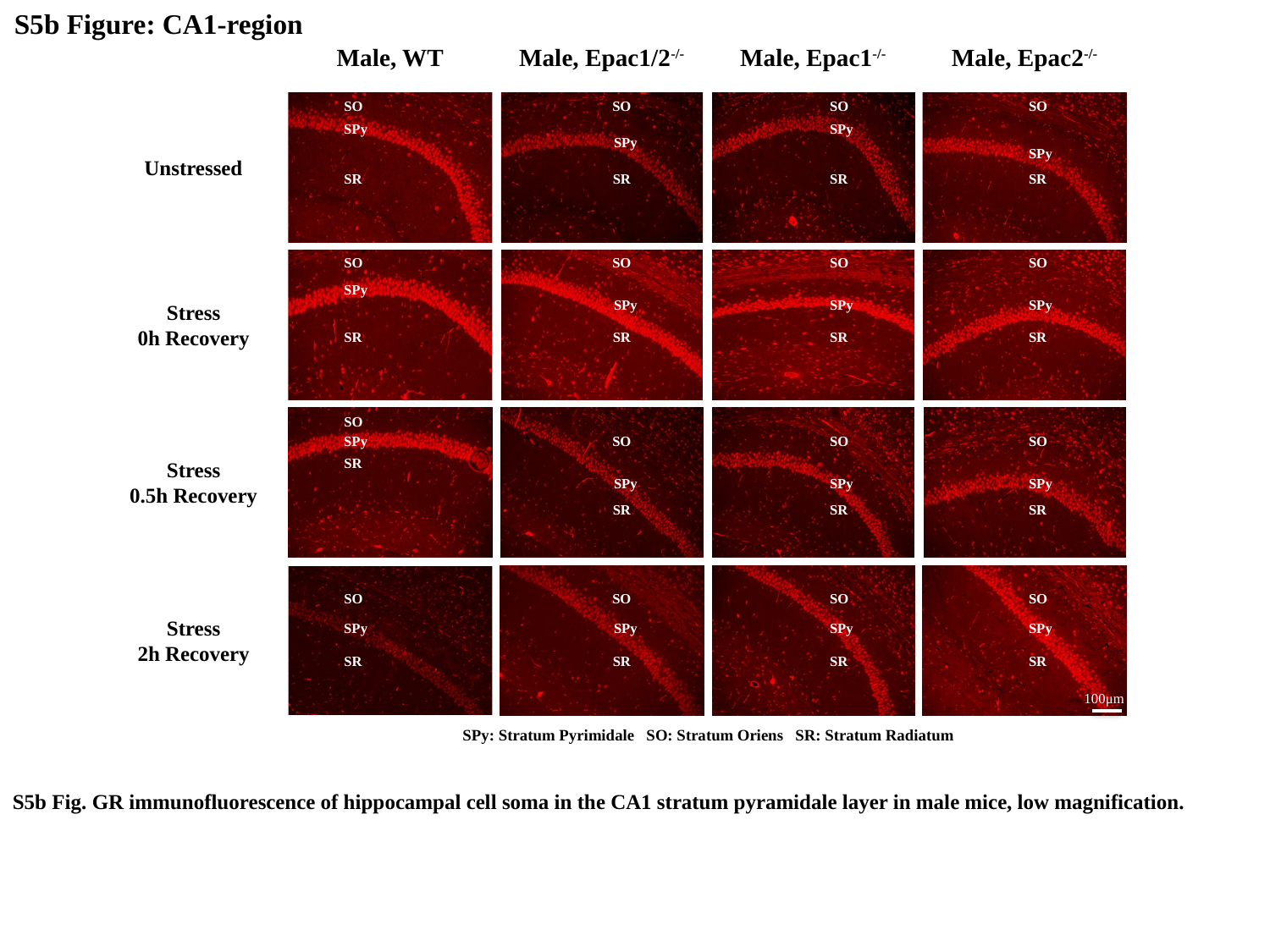

S5b Figure: CA1-region
Male, WT
Male, Epac1/2-/-
Male, Epac1-/-
Male, Epac2-/-
SO
SO
SO
SO
SPy
SPy
SPy
SPy
Unstressed
SR
SR
SR
SR
SO
SO
SO
SO
SPy
SPy
SPy
SPy
Stress
0h Recovery
SR
SR
SR
SR
SO
SPy
SO
SO
SO
SR
Stress
0.5h Recovery
SPy
SPy
SPy
SR
SR
SR
SO
SO
SO
SO
Stress
2h Recovery
SPy
SPy
SPy
SPy
SR
SR
SR
SR
100μm
SPy: Stratum Pyrimidale SO: Stratum Oriens SR: Stratum Radiatum
S5b Fig. GR immunofluorescence of hippocampal cell soma in the CA1 stratum pyramidale layer in male mice, low magnification.

## Slide 3
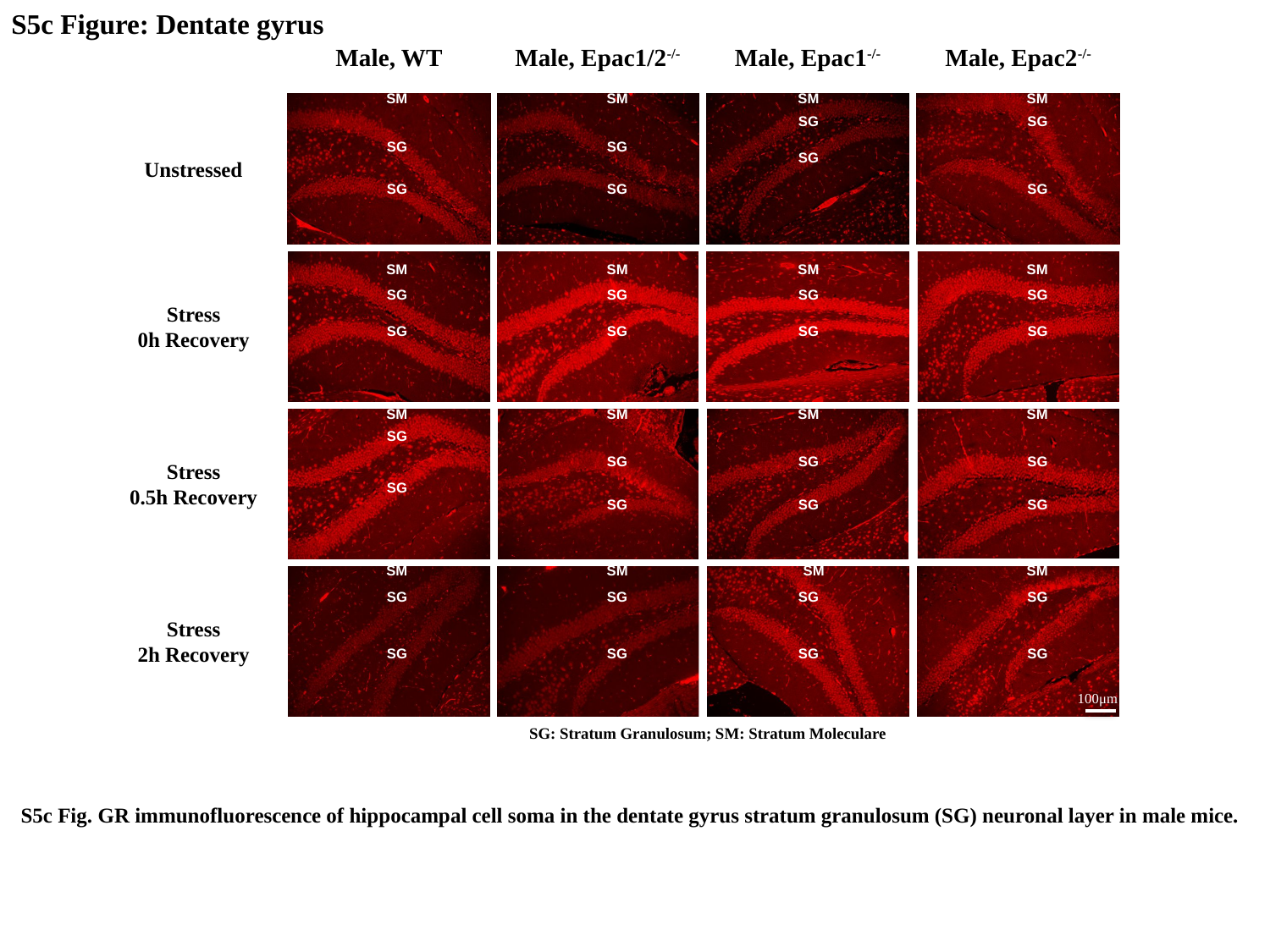

S5c Figure: Dentate gyrus
Male, WT
Male, Epac1/2-/-
Male, Epac1-/-
Male, Epac2-/-
SM
SM
SM
SM
SG
SG
SG
SG
SG
Unstressed
SG
SG
SG
SM
SM
SM
SM
SG
SG
SG
SG
Stress
0h Recovery
SG
SG
SG
SG
SM
SM
SM
SM
SG
SG
SG
SG
Stress
0.5h Recovery
SG
SG
SG
SG
SM
SM
SM
SM
SG
SG
SG
SG
Stress
2h Recovery
SG
SG
SG
SG
100μm
SG: Stratum Granulosum; SM: Stratum Moleculare
S5c Fig. GR immunofluorescence of hippocampal cell soma in the dentate gyrus stratum granulosum (SG) neuronal layer in male mice.

## Slide 4
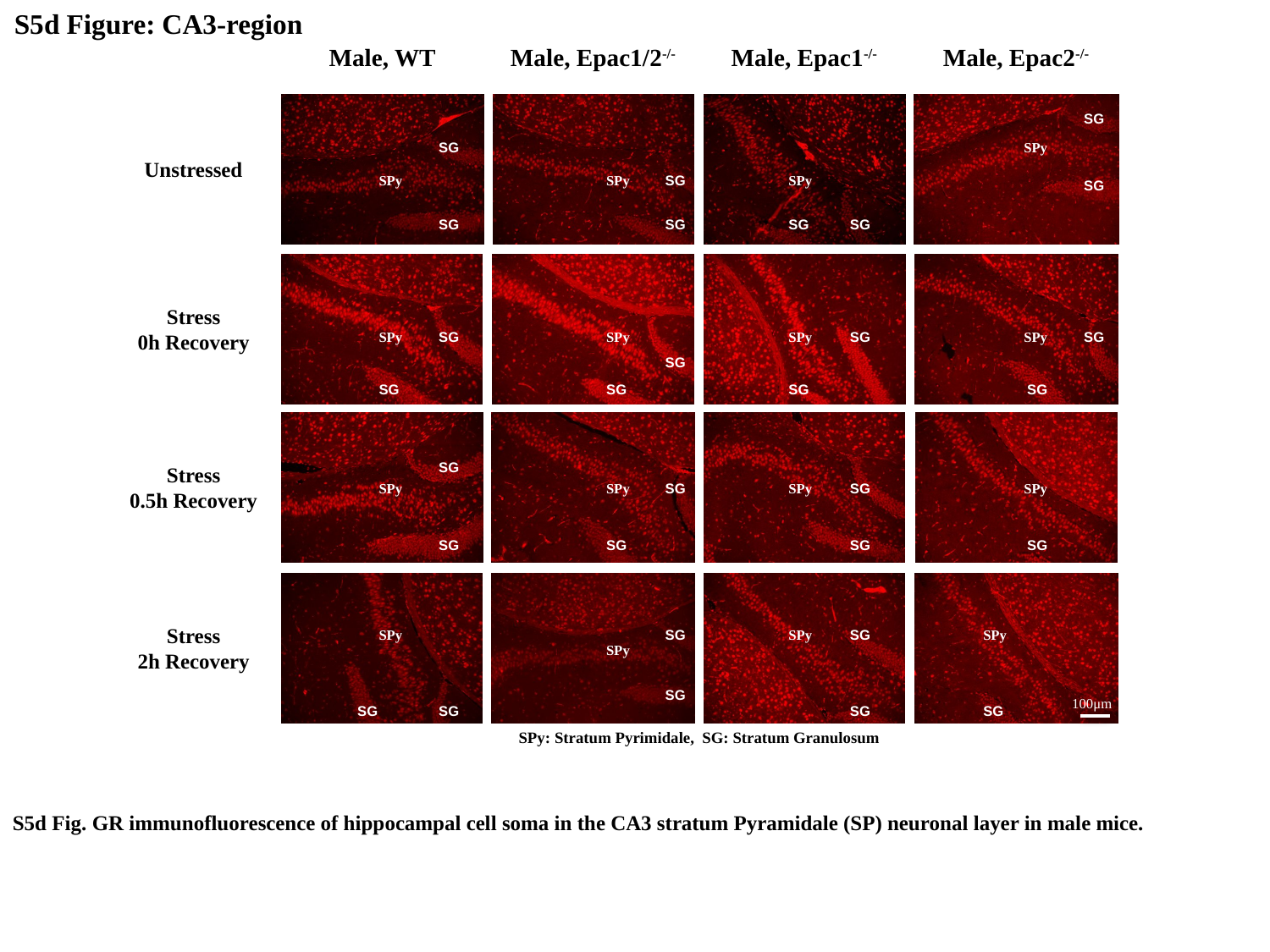

S5d Figure: CA3-region
Male, WT
Male, Epac1/2-/-
Male, Epac1-/-
Male, Epac2-/-
SG
SG
SPy
Unstressed
SPy
SPy
SG
SPy
SG
SG
SG
SG
SG
Stress
0h Recovery
SPy
SG
SPy
SPy
SG
SPy
SG
SG
SG
SG
SG
SG
SG
Stress
0.5h Recovery
SPy
SPy
SG
SPy
SG
SPy
SG
SG
SG
SG
Stress
2h Recovery
SPy
SG
SPy
SG
SPy
SPy
SG
100μm
SG
SG
SG
SG
SPy: Stratum Pyrimidale, SG: Stratum Granulosum
S5d Fig. GR immunofluorescence of hippocampal cell soma in the CA3 stratum Pyramidale (SP) neuronal layer in male mice.
