# Supplementary material for: Deletion of exchange proteins directly activated by cAMP (Epac) causes defects in hippocampal signaling in female mice
Source: PLoS One. 2018 Jul 26;13(7):e0200935. doi: 10.1371/journal.pone.0200935 (PMC6062027; doi:10.1371/journal.pone.0200935)
Supplement: S6 Fig — Wt, Epac1/2-/-, Epac1-/- or Epac2-/- mice were kept at standard housing conditions (unstressed) or exposed to 30min of restraint stress, and either culled immediately after the stressor (0h recovery), or after recovery from the stress for 30min or 2h. Paraffin-embedded coronal brain sections (15μm) were stained with a GR-specific antibody and thereafter visualized under a 60X objective of the Nikon Te 2000-e microscope with a TRITC fluorescent light filter, and captured with a Nikon Digital Sight DS-U1 camera. The 10μm scale bar in the lower right panel applies to all images shown in the figure. As shown in the figure, GR immunofluorescence staining was confined to the nuclear regions of the hippocampal cell soma. (PPTX) [file pone.0200935.s006.pptx]

## Slide 1
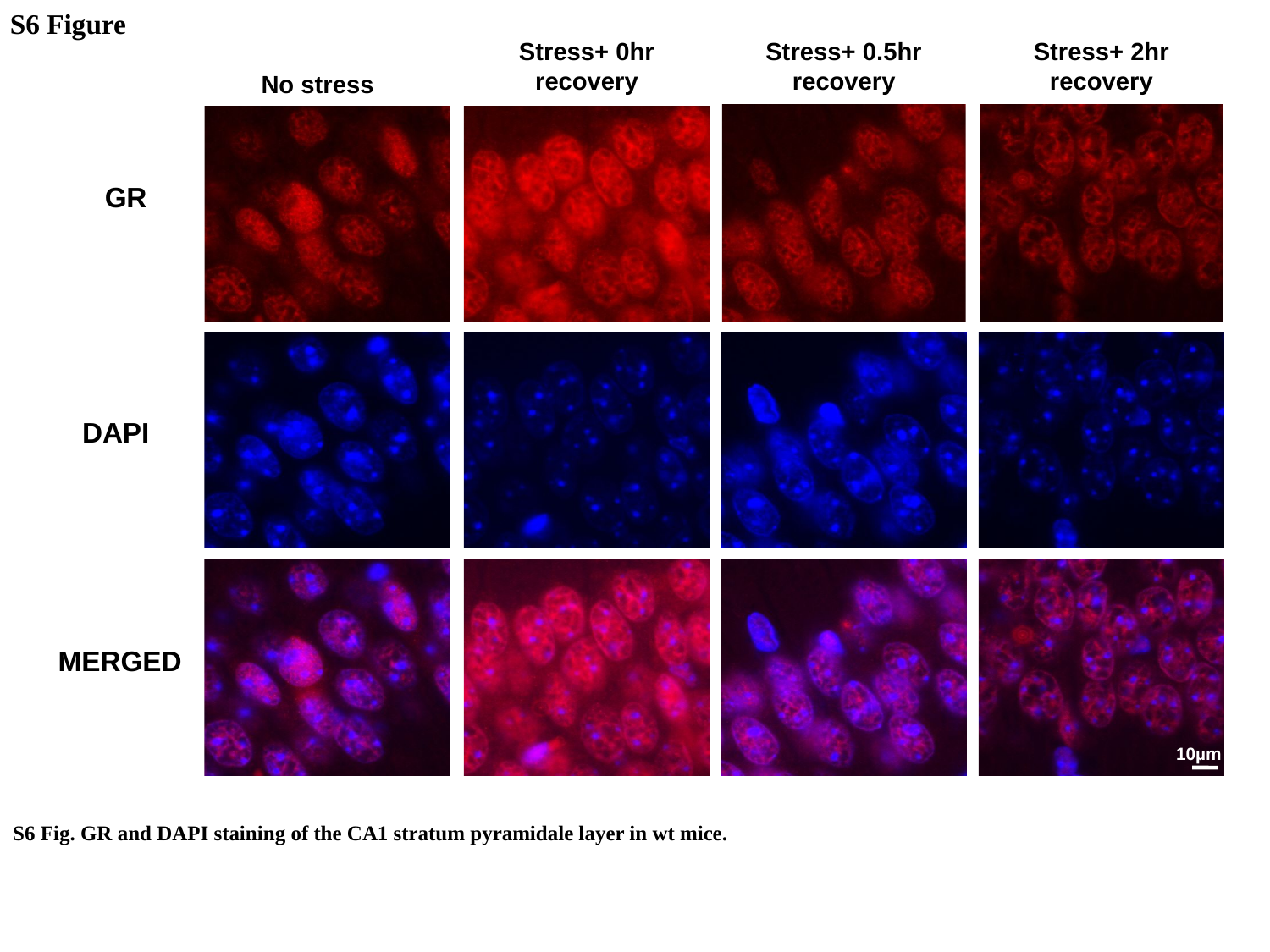

S6 Figure
Stress+ 0hr recovery
Stress+ 0.5hr recovery
Stress+ 2hr recovery
No stress
GR
DAPI
MERGED
10µm
S6 Fig. GR and DAPI staining of the CA1 stratum pyramidale layer in wt mice.
