# Supplementary material for: Deletion of exchange proteins directly activated by cAMP (Epac) causes defects in hippocampal signaling in female mice
Source: PLoS One. 2018 Jul 26;13(7):e0200935. doi: 10.1371/journal.pone.0200935 (PMC6062027; doi:10.1371/journal.pone.0200935)
Supplement: S8 Fig — Female (A) and male (B) mice were kept at standard housing conditions (-) or exposed to 30min of restraint stress (+). The hippocampus was dissected out after the mice had been culled with CO2 immediately after the stressor (0h), or after recovery from the stress for 30min or 2h, and mRNA prepared. qPCR analyses were performed to determine Ngfi-A mRNA levels. The qPCR values were normalized to the expression of the housekeeping genes Sdha and Ppib, and are shown as average of relative fold change, ± SEM of three independent experiments performed in triplicates (n = 7–9). Two-way ANOVA with Tukey’s adjustment for multiple comparisons was used for statistical analysis. F-statistics (F(Dfn, DFd)) for the female group: Interaction: F(9, 128) = 6.755, p<0.0001 and the male group: Interaction: F(9, 118) = 3.383, p = 0.0010. (PPTX) [file pone.0200935.s008.pptx]

## Slide 1
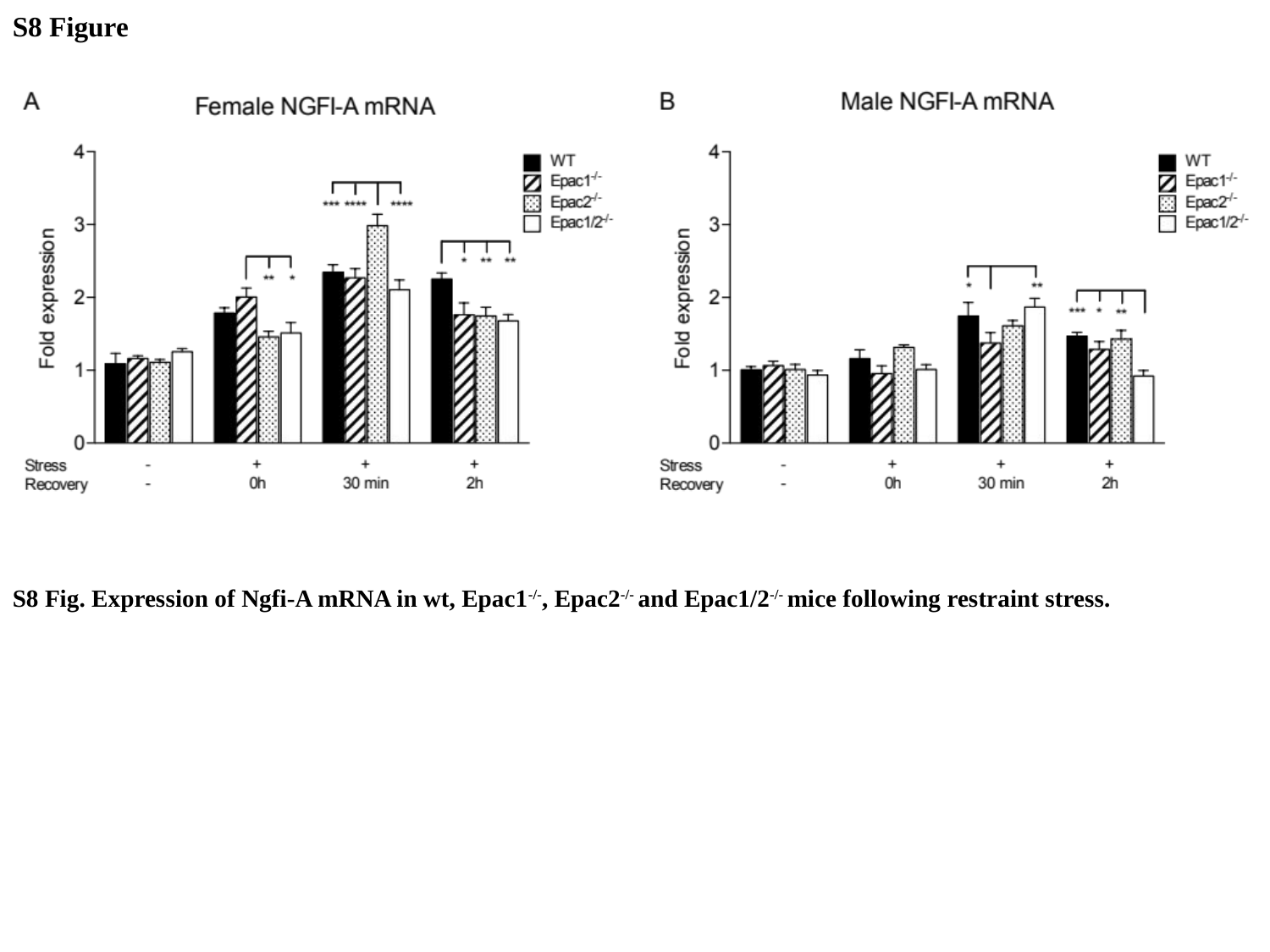

S8 Figure
S8 Fig. Expression of Ngfi-A mRNA in wt, Epac1-/-, Epac2-/- and Epac1/2-/- mice following restraint stress.
