# Supplementary material for: Deletion of exchange proteins directly activated by cAMP (Epac) causes defects in hippocampal signaling in female mice
Source: PLoS One. 2018 Jul 26;13(7):e0200935. doi: 10.1371/journal.pone.0200935 (PMC6062027; doi:10.1371/journal.pone.0200935)
Supplement: S1 Table — (PPTX) [file pone.0200935.s009.pptx]

## Slide 1
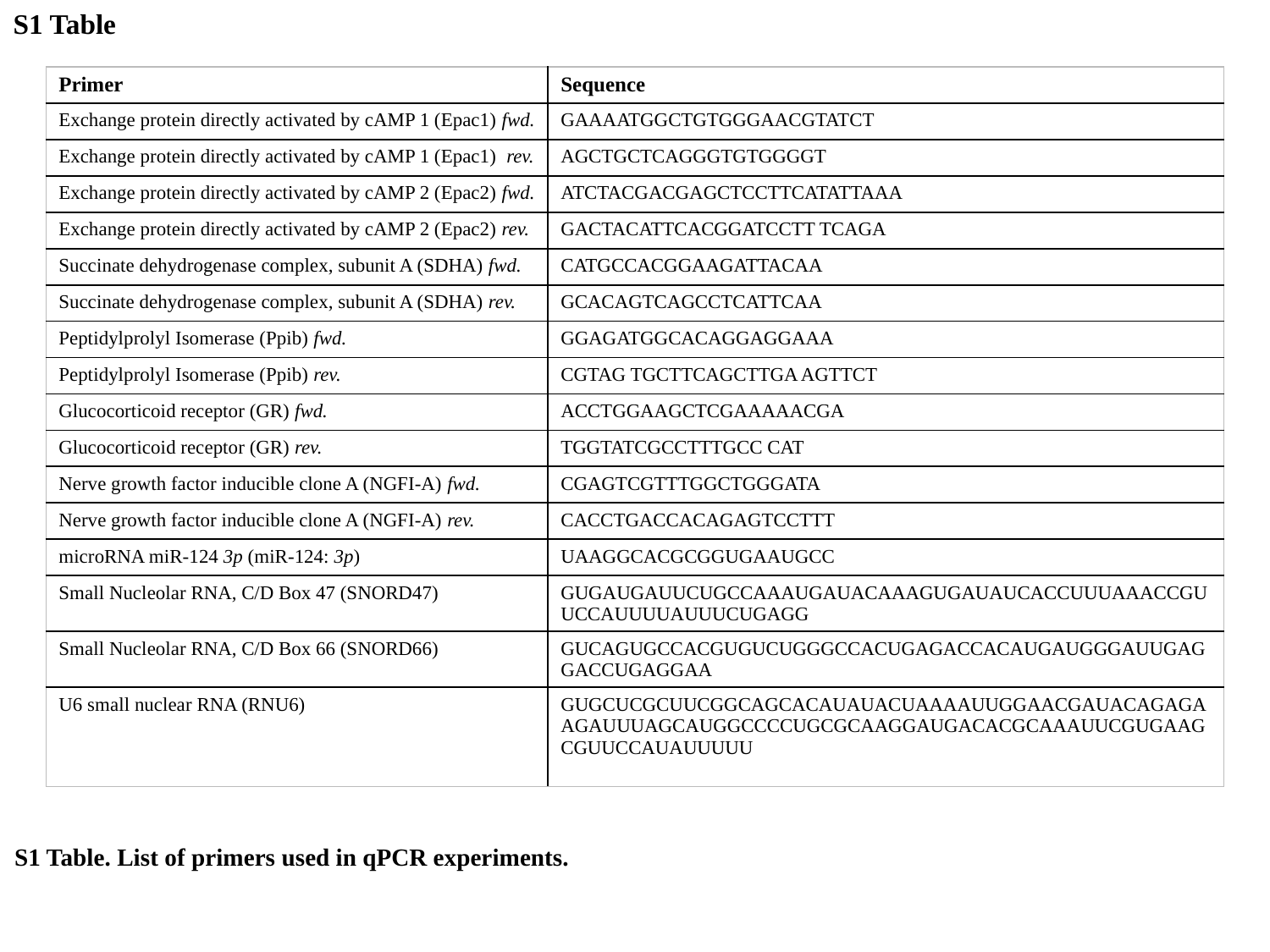

S1 Table
| Primer | Sequence |
| --- | --- |
| Exchange protein directly activated by cAMP 1 (Epac1) fwd. | GAAAATGGCTGTGGGAACGTATCT |
| Exchange protein directly activated by cAMP 1 (Epac1) rev. | AGCTGCTCAGGGTGTGGGGT |
| Exchange protein directly activated by cAMP 2 (Epac2) fwd. | ATCTACGACGAGCTCCTTCATATTAAA |
| Exchange protein directly activated by cAMP 2 (Epac2) rev. | GACTACATTCACGGATCCTT TCAGA |
| Succinate dehydrogenase complex, subunit A (SDHA) fwd. | CATGCCACGGAAGATTACAA |
| Succinate dehydrogenase complex, subunit A (SDHA) rev. | GCACAGTCAGCCTCATTCAA |
| Peptidylprolyl Isomerase (Ppib) fwd. | GGAGATGGCACAGGAGGAAA |
| Peptidylprolyl Isomerase (Ppib) rev. | CGTAG TGCTTCAGCTTGA AGTTCT |
| Glucocorticoid receptor (GR) fwd. | ACCTGGAAGCTCGAAAAACGA |
| Glucocorticoid receptor (GR) rev. | TGGTATCGCCTTTGCC CAT |
| Nerve growth factor inducible clone A (NGFI-A) fwd. | CGAGTCGTTTGGCTGGGATA |
| Nerve growth factor inducible clone A (NGFI-A) rev. | CACCTGACCACAGAGTCCTTT |
| microRNA miR-124 3p (miR-124: 3p) | UAAGGCACGCGGUGAAUGCC |
| Small Nucleolar RNA, C/D Box 47 (SNORD47) | GUGAUGAUUCUGCCAAAUGAUACAAAGUGAUAUCACCUUUAAACCGUUCCAUUUUAUUUCUGAGG |
| Small Nucleolar RNA, C/D Box 66 (SNORD66) | GUCAGUGCCACGUGUCUGGGCCACUGAGACCACAUGAUGGGAUUGAGGACCUGAGGAA |
| U6 small nuclear RNA (RNU6) | GUGCUCGCUUCGGCAGCACAUAUACUAAAAUUGGAACGAUACAGAGAAGAUUUAGCAUGGCCCCUGCGCAAGGAUGACACGCAAAUUCGUGAAGCGUUCCAUAUUUUU |
S1 Table. List of primers used in qPCR experiments.
