# Supplementary material for: Deletion of exchange proteins directly activated by cAMP (Epac) causes defects in hippocampal signaling in female mice
Source: PLoS One. 2018 Jul 26;13(7):e0200935. doi: 10.1371/journal.pone.0200935 (PMC6062027; doi:10.1371/journal.pone.0200935)
Supplement: S2 Table — Based on the results shown in Fig 1, serum corticosterone levels of unstressed (-) and stressed (0h, 30min and 2h) mice (shown as mean ± SD) were compared and significance determined by Two-way ANOVA with Tukey's adjustment for multiple comparisons. Statistical analyses were performed separately for the female and male groups. aap<0.01, aaap<0.001 and aaaap<0.0001 unstressed mice (-) compared to mice subjected to 30min stress with recovery (0h, 30min or 2h), same genotype and sex. bp<0.5, bbbp<0.001 and bbbbp<0.0001 mice subjected to 30min stress, no recovery compared to mice subjected to 30min stress with recovery (30min or 2h). cp<0.5 and cccp<0.001 mice subjected to 30min stress with 30min recovery compared to mice subjected to 30min stress with 2h recovery, same genotype and sex. n = 7–9 mice per group. F-statistics (F(Dfn, DFd)) for the female group: Interaction: F(9,102) = 3.713, p = 0.0005 and the male group: Interaction: F(9,108) = 1.759, p = 0.0846. (PPTX) [file pone.0200935.s010.pptx]

## Slide 1
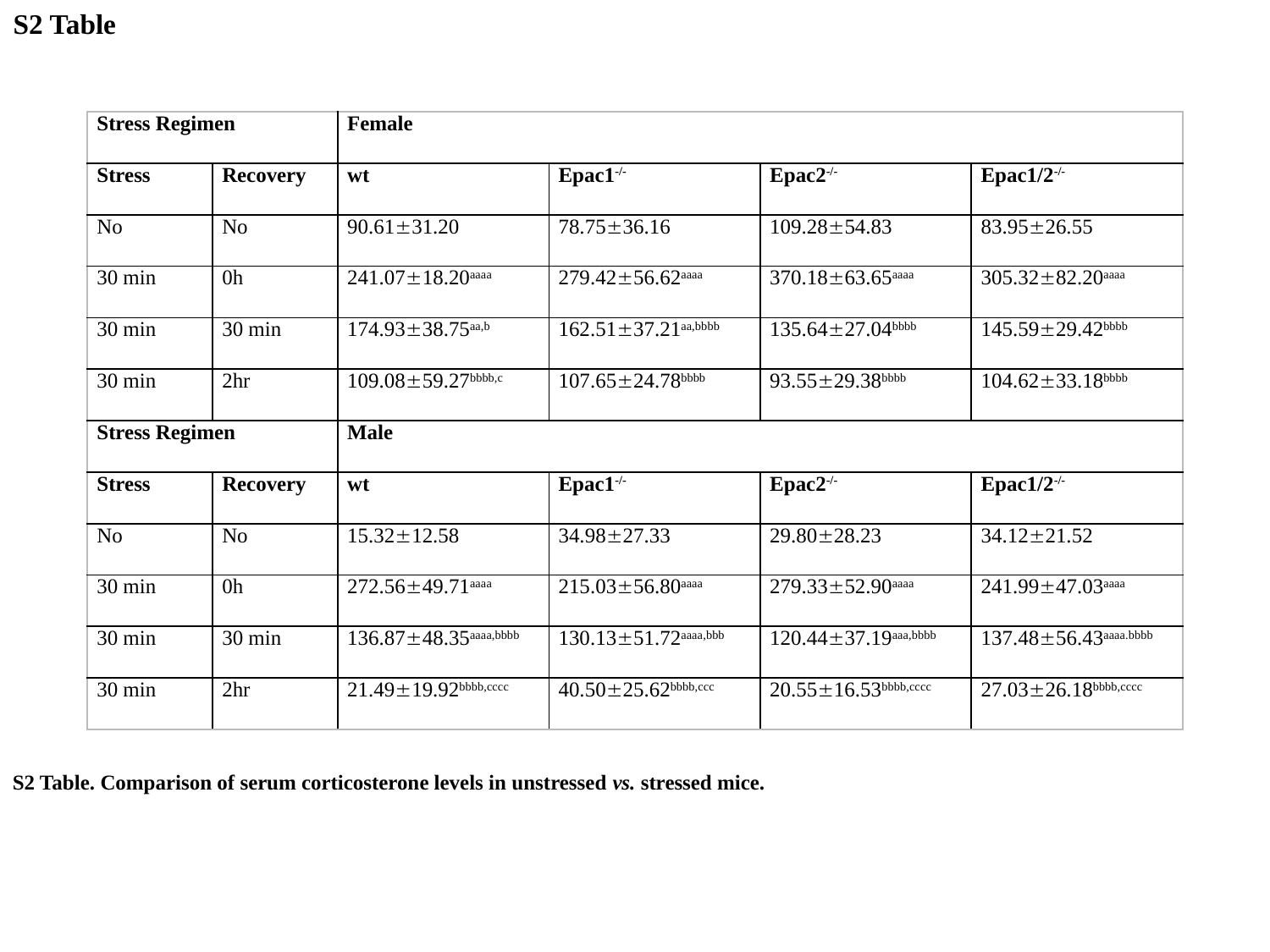

S2 Table
| Stress Regimen | | Female | | | |
| --- | --- | --- | --- | --- | --- |
| Stress | Recovery | wt | Epac1-/- | Epac2-/- | Epac1/2-/- |
| No | No | 90.61±31.20 | 78.75±36.16 | 109.28±54.83 | 83.95±26.55 |
| 30 min | 0h | 241.07±18.20aaaa | 279.42±56.62aaaa | 370.18±63.65aaaa | 305.32±82.20aaaa |
| 30 min | 30 min | 174.93±38.75aa,b | 162.51±37.21aa,bbbb | 135.64±27.04bbbb | 145.59±29.42bbbb |
| 30 min | 2hr | 109.08±59.27bbbb,c | 107.65±24.78bbbb | 93.55±29.38bbbb | 104.62±33.18bbbb |
| Stress Regimen | | Male | | | |
| Stress | Recovery | wt | Epac1-/- | Epac2-/- | Epac1/2-/- |
| No | No | 15.32±12.58 | 34.98±27.33 | 29.80±28.23 | 34.12±21.52 |
| 30 min | 0h | 272.56±49.71aaaa | 215.03±56.80aaaa | 279.33±52.90aaaa | 241.99±47.03aaaa |
| 30 min | 30 min | 136.87±48.35aaaa,bbbb | 130.13±51.72aaaa,bbb | 120.44±37.19aaa,bbbb | 137.48±56.43aaaa.bbbb |
| 30 min | 2hr | 21.49±19.92bbbb,cccc | 40.50±25.62bbbb,ccc | 20.55±16.53bbbb,cccc | 27.03±26.18bbbb,cccc |
S2 Table. Comparison of serum corticosterone levels in unstressed vs. stressed mice.
