# Supplementary material for: Deletion of exchange proteins directly activated by cAMP (Epac) causes defects in hippocampal signaling in female mice
Source: PLoS One. 2018 Jul 26;13(7):e0200935. doi: 10.1371/journal.pone.0200935 (PMC6062027; doi:10.1371/journal.pone.0200935)
Supplement: S3 Table — Based on the results shown in Fig 3, GR staining in unstressed female and male mice (all genotypes, presented as average ±SD gray values) were compared and significance determined by One-way ANOVA with Tukey`s adjustment for multiple comparisons. Statistical analyses were performed separately for each region. ap≤0.05, aap≤0.01,aaap<0.001 and aaaap≤0.0001 unstressed (-) female wt mice compared to unstressed female Epac1-/-, Epac2-/-, Epac1/2-/- mice and male mice (all genotypes), same region, bp≤0.05, bbp≤0.01,bbbp<0.001 and bbbbp≤0.0001 unstressed female Epac1-/- mice compared to unstressed female Epac2-/-, Epac1/2-/- mice and male mice (all genotypes), same region. cccp<0.001 and ccccp≤0.0001 unstressed female Epac2-/- mice compared to unstressed female Epac1/2-/- mice and male mice (all genotypes), same region. ddp≤0.01, dddp<0.001 and ddddp≤0.0001 unstressed female Epac1/2-/- mice compared to male mice (all genotypes), same region. ep≤0.05, eep≤0.01 and eeep<0.001 unstressed male wt mice compared to unstressed male Epac1-/-, Epac2-/- and Epac1/2-/- mice, same region. n = 3 mice per group, and for each mouse, 3 sections of the hippocampus where quantified for GR staining. F-statistics (F(DFn, DFd)) DG; Interaction: F(7, 64) = 20.88, p<0.0001, CA1; Interaction: F(7, 64) = 25.46, p<0.0001 and CA3; Interaction: F(7, 64) = 8.804, p<0.0001. (PPTX) [file pone.0200935.s011.pptx]

## Slide 1
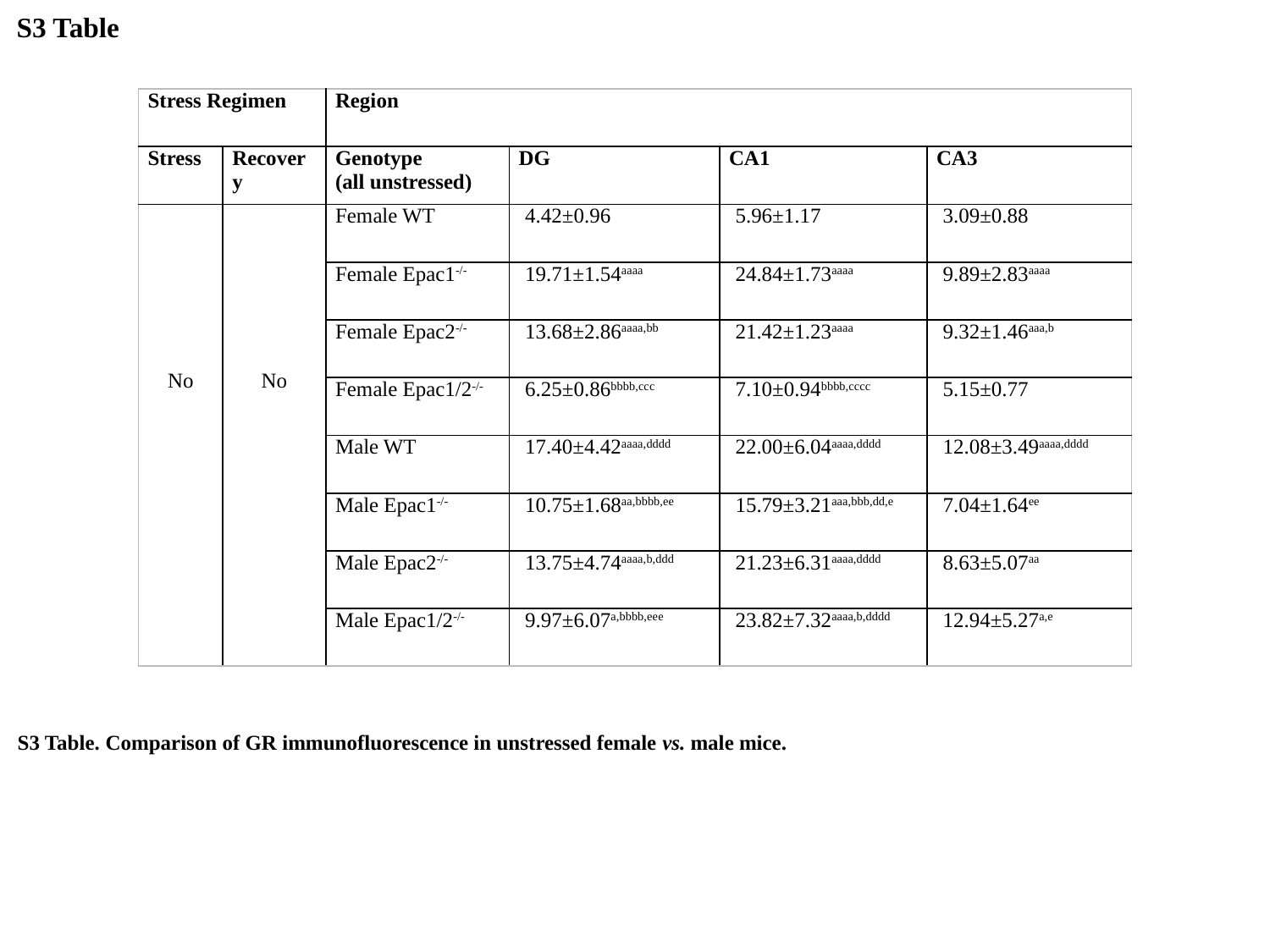

S3 Table
| Stress Regimen | | Region | | | |
| --- | --- | --- | --- | --- | --- |
| Stress | Recovery | Genotype (all unstressed) | DG | CA1 | CA3 |
| No | No | Female WT | 4.42±0.96 | 5.96±1.17 | 3.09±0.88 |
| | | Female Epac1-/- | 19.71±1.54aaaa | 24.84±1.73aaaa | 9.89±2.83aaaa |
| | | Female Epac2-/- | 13.68±2.86aaaa,bb | 21.42±1.23aaaa | 9.32±1.46aaa,b |
| | | Female Epac1/2-/- | 6.25±0.86bbbb,ccc | 7.10±0.94bbbb,cccc | 5.15±0.77 |
| | | Male WT | 17.40±4.42aaaa,dddd | 22.00±6.04aaaa,dddd | 12.08±3.49aaaa,dddd |
| | | Male Epac1-/- | 10.75±1.68aa,bbbb,ee | 15.79±3.21aaa,bbb,dd,e | 7.04±1.64ee |
| | | Male Epac2-/- | 13.75±4.74aaaa,b,ddd | 21.23±6.31aaaa,dddd | 8.63±5.07aa |
| | | Male Epac1/2-/- | 9.97±6.07a,bbbb,eee | 23.82±7.32aaaa,b,dddd | 12.94±5.27a,e |
S3 Table. Comparison of GR immunofluorescence in unstressed female vs. male mice.
