# Supplementary material for: Deletion of exchange proteins directly activated by cAMP (Epac) causes defects in hippocampal signaling in female mice
Source: PLoS One. 2018 Jul 26;13(7):e0200935. doi: 10.1371/journal.pone.0200935 (PMC6062027; doi:10.1371/journal.pone.0200935)
Supplement: S4 Table — Based on the results shown in S7 Fig, GR mRNA levels in unstressed (-) and stressed (0h, 30min and 2h) mice were compared and significance determined by Two-way ANOVA The data is presented as average of relative fold change ± SEM of three independent experiments performed in triplicates (n = 7–9). Statistical analyses were performed separately for the female and male groups. No significant differences were found. F-statistics (F(Dfn, DFd)) for the female group: Interaction: F(9, 113) = 0.3331, p = 0.9623 and the male group: Interaction: F(9, 119) = 0.5079, p = 0.8664. (PPTX) [file pone.0200935.s012.pptx]

## Slide 1
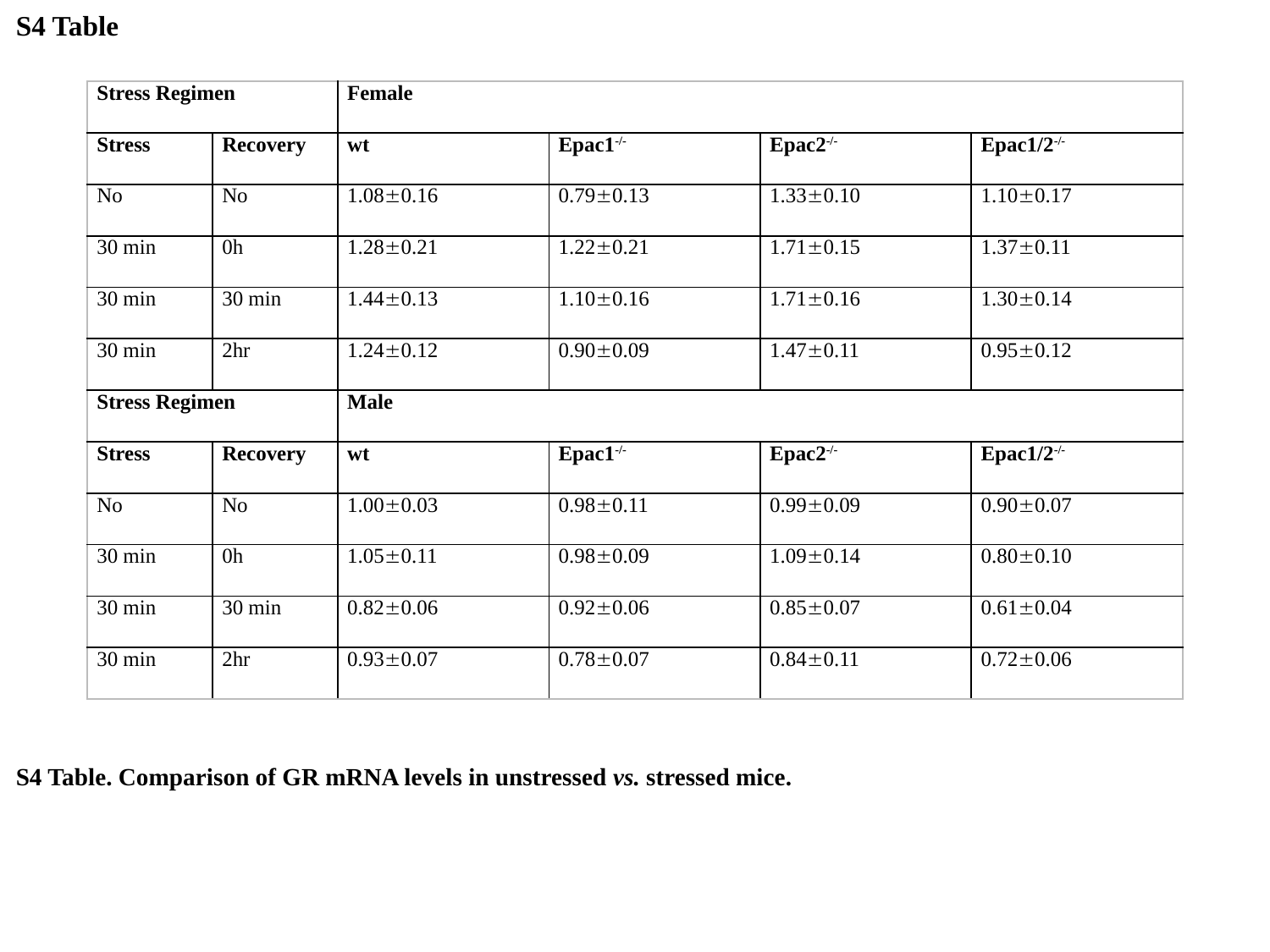

S4 Table
| Stress Regimen | | Female | | | |
| --- | --- | --- | --- | --- | --- |
| Stress | Recovery | wt | Epac1-/- | Epac2-/- | Epac1/2-/- |
| No | No | 1.08±0.16 | 0.79±0.13 | 1.33±0.10 | 1.10±0.17 |
| 30 min | 0h | 1.28±0.21 | 1.22±0.21 | 1.71±0.15 | 1.37±0.11 |
| 30 min | 30 min | 1.44±0.13 | 1.10±0.16 | 1.71±0.16 | 1.30±0.14 |
| 30 min | 2hr | 1.24±0.12 | 0.90±0.09 | 1.47±0.11 | 0.95±0.12 |
| Stress Regimen | | Male | | | |
| Stress | Recovery | wt | Epac1-/- | Epac2-/- | Epac1/2-/- |
| No | No | 1.00±0.03 | 0.98±0.11 | 0.99±0.09 | 0.90±0.07 |
| 30 min | 0h | 1.05±0.11 | 0.98±0.09 | 1.09±0.14 | 0.80±0.10 |
| 30 min | 30 min | 0.82±0.06 | 0.92±0.06 | 0.85±0.07 | 0.61±0.04 |
| 30 min | 2hr | 0.93±0.07 | 0.78±0.07 | 0.84±0.11 | 0.72±0.06 |
S4 Table. Comparison of GR mRNA levels in unstressed vs. stressed mice.
