# Supplementary material for: Deletion of exchange proteins directly activated by cAMP (Epac) causes defects in hippocampal signaling in female mice
Source: PLoS One. 2018 Jul 26;13(7):e0200935. doi: 10.1371/journal.pone.0200935 (PMC6062027; doi:10.1371/journal.pone.0200935)
Supplement: S5 Table — Based on the results shown in Fig 4, miR-124 levels in unstressed (-) and stressed (0h, 30min and 2h) mice were compared and significance determined by Two-way ANOVA with Tukey's adjustment for multiple comparisons. The data is presented as average of relative fold change ± SEM of three independent experiments performed in triplicates (n = 7–9). Statistical analyses were performed separately for the female and male groups. ap<0.05 aap<0.01 and aaaap<0.0001 unstressed mice (-) compared to mice subjected to 30min stress with recovery (0h, 30min or 2h), same genotype and sex. bbbbp<0.0001 mice subjected to 30min stress, no recovery compared to mice subjected to 30min stress with recovery (30min or 2h), same genotype and sex. ccccp<0.0001 mice subjected to 30min stress with 30min recovery compared to mice subjected to 30min stress and 2h recovery, same genotype and sex. F-statistics (F(Dfn, DFd)) for the female group: Interaction: F(9, 122) = 4.359, p<0.0001 and the male group: Interaction: F(9, 128) = 12.90, p<0.0001. (PPTX) [file pone.0200935.s013.pptx]

## Slide 1
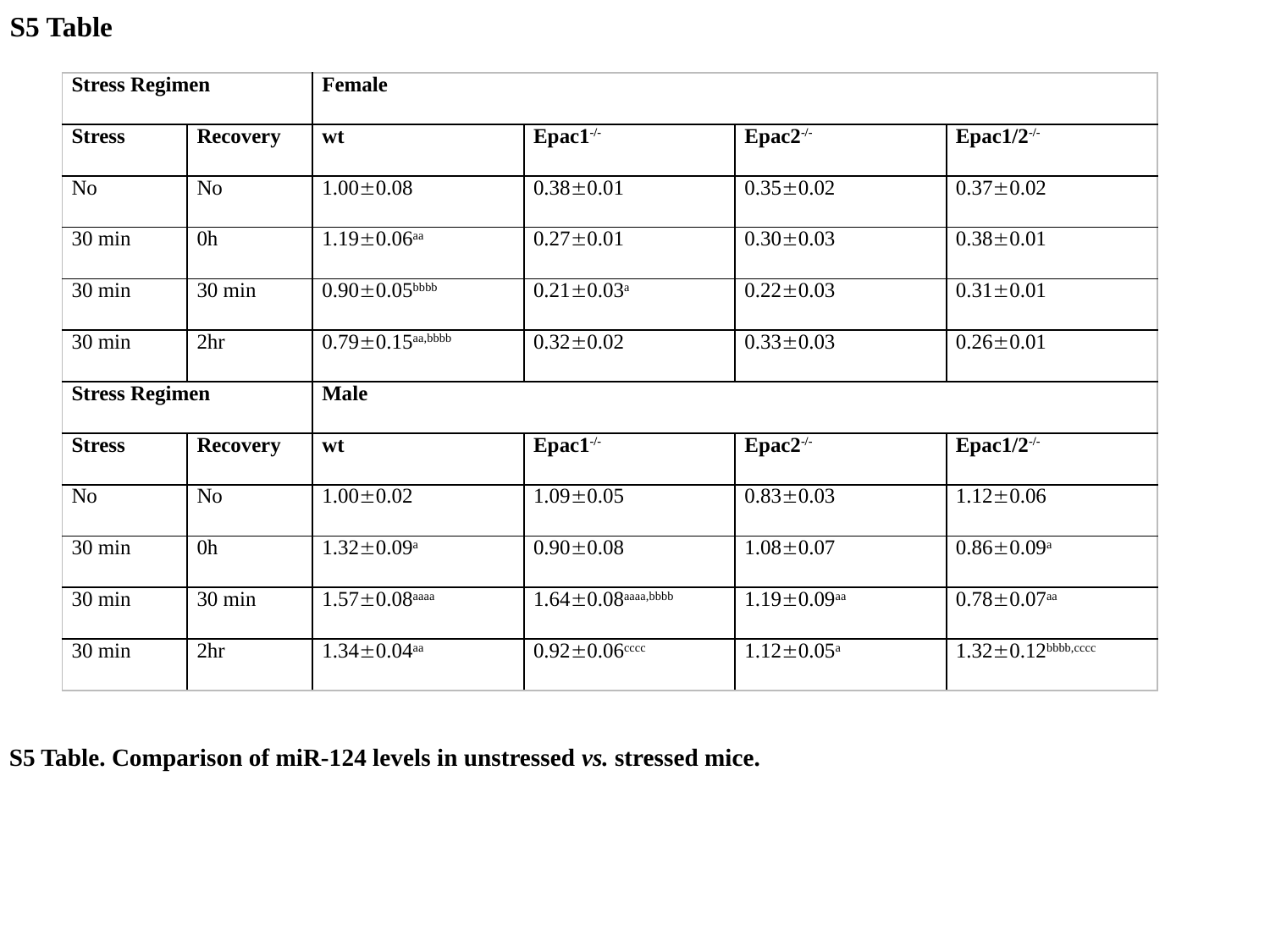

S5 Table
| Stress Regimen | | Female | | | |
| --- | --- | --- | --- | --- | --- |
| Stress | Recovery | wt | Epac1-/- | Epac2-/- | Epac1/2-/- |
| No | No | 1.00±0.08 | 0.38±0.01 | 0.35±0.02 | 0.37±0.02 |
| 30 min | 0h | 1.19±0.06aa | 0.27±0.01 | 0.30±0.03 | 0.38±0.01 |
| 30 min | 30 min | 0.90±0.05bbbb | 0.21±0.03a | 0.22±0.03 | 0.31±0.01 |
| 30 min | 2hr | 0.79±0.15aa,bbbb | 0.32±0.02 | 0.33±0.03 | 0.26±0.01 |
| Stress Regimen | | Male | | | |
| Stress | Recovery | wt | Epac1-/- | Epac2-/- | Epac1/2-/- |
| No | No | 1.00±0.02 | 1.09±0.05 | 0.83±0.03 | 1.12±0.06 |
| 30 min | 0h | 1.32±0.09a | 0.90±0.08 | 1.08±0.07 | 0.86±0.09a |
| 30 min | 30 min | 1.57±0.08aaaa | 1.64±0.08aaaa,bbbb | 1.19±0.09aa | 0.78±0.07aa |
| 30 min | 2hr | 1.34±0.04aa | 0.92±0.06cccc | 1.12±0.05a | 1.32±0.12bbbb,cccc |
S5 Table. Comparison of miR-124 levels in unstressed vs. stressed mice.
