# Supplementary material for: Deletion of exchange proteins directly activated by cAMP (Epac) causes defects in hippocampal signaling in female mice
Source: PLoS One. 2018 Jul 26;13(7):e0200935. doi: 10.1371/journal.pone.0200935 (PMC6062027; doi:10.1371/journal.pone.0200935)
Supplement: S6 Table — Based on the results shown in S8 Fig, Ngfi-A mRNA levels in unstressed (-) and stressed (0h, 30min and 2h) mice were compared and significance determined by Two-way ANOVA with Tukey's adjustment for multiple comparisons. The data is presented as average of relative fold change ± SEM of three independent experiments performed in triplicates (n = 7–9). Statistical analyses were performed separately for the female and male groups. ap<0.05 aap<0.01, aaap<0.001 and aaaap<0.0001 unstressed mice (-) compared to mice subjected to 30min stress with recovery (0h, 30min or 2h), same genotype and sex. bp<0.05, bbp<0.01, bbbp<0.001 and bbbbp<0.0001 mice subjected to 30min stress, no recovery compared to mice subjected to 30min stress with recovery (30min or 2h), same genotype and sex. cp<0.05, ccp<0.01 and ccccp<0.0001 mice subjected to 30min stress with 30min recovery compared to mice subjected to 30min stress with 2h recovery. F-statistics (F(Dfn, DFd)) for the female group: Interaction: F(9, 128) = 6.755, p<0.0001 and the male group: Interaction: F(9, 118) = 3.383, p = 0.0010. (PPTX) [file pone.0200935.s014.pptx]

## Slide 1
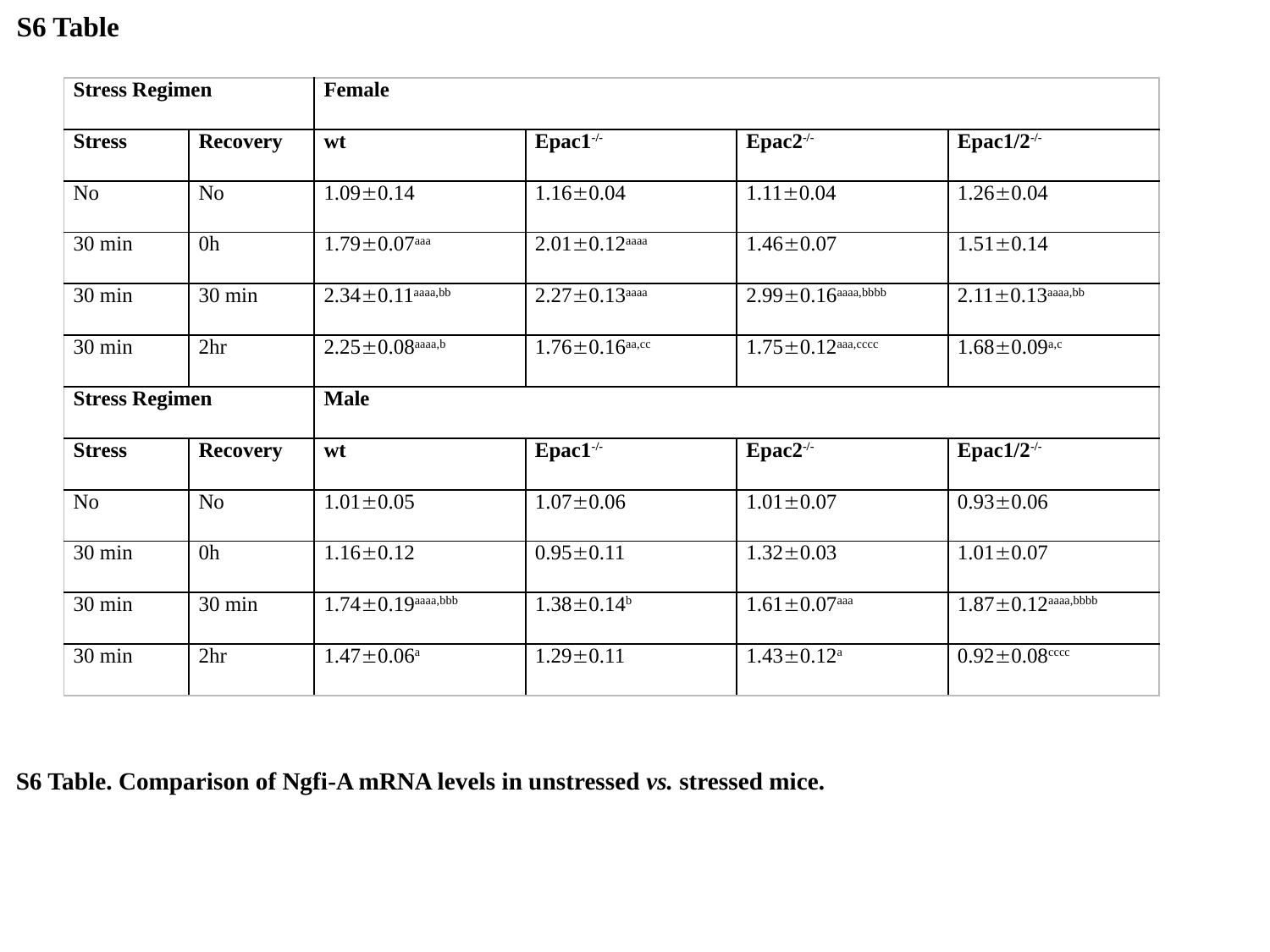

S6 Table
| Stress Regimen | | Female | | | |
| --- | --- | --- | --- | --- | --- |
| Stress | Recovery | wt | Epac1-/- | Epac2-/- | Epac1/2-/- |
| No | No | 1.09±0.14 | 1.16±0.04 | 1.11±0.04 | 1.26±0.04 |
| 30 min | 0h | 1.79±0.07aaa | 2.01±0.12aaaa | 1.46±0.07 | 1.51±0.14 |
| 30 min | 30 min | 2.34±0.11aaaa,bb | 2.27±0.13aaaa | 2.99±0.16aaaa,bbbb | 2.11±0.13aaaa,bb |
| 30 min | 2hr | 2.25±0.08aaaa,b | 1.76±0.16aa,cc | 1.75±0.12aaa,cccc | 1.68±0.09a,c |
| Stress Regimen | | Male | | | |
| Stress | Recovery | wt | Epac1-/- | Epac2-/- | Epac1/2-/- |
| No | No | 1.01±0.05 | 1.07±0.06 | 1.01±0.07 | 0.93±0.06 |
| 30 min | 0h | 1.16±0.12 | 0.95±0.11 | 1.32±0.03 | 1.01±0.07 |
| 30 min | 30 min | 1.74±0.19aaaa,bbb | 1.38±0.14b | 1.61±0.07aaa | 1.87±0.12aaaa,bbbb |
| 30 min | 2hr | 1.47±0.06a | 1.29±0.11 | 1.43±0.12a | 0.92±0.08cccc |
S6 Table. Comparison of Ngfi-A mRNA levels in unstressed vs. stressed mice.
